# Molecular Basis of Ligand-Dependent Regulation of NadR, the Transcriptional Repressor of Meningococcal Virulence Factor NadA

**DOI:** 10.1371/journal.ppat.1005557

**Published:** 2016-04-22

**Authors:** Alessia Liguori, Enrico Malito, Paola Lo Surdo, Luca Fagnocchi, Francesca Cantini, Andreas F. Haag, Sébastien Brier, Mariagrazia Pizza, Isabel Delany, Matthew J. Bottomley

**Affiliations:** 1 GSK Vaccines Srl, Siena, Italy; 2 Istituto Nazionale di Genetica Molecolare “Romeo ed Enrica Invernizzi”, Milan, Italy; 3 CERM Magnetic Resonance Centre, University of Florence, Florence, Italy; 4 Institute of Infection, Immunity & Inflammation, University of Glasgow, Glasgow, United Kingdom; 5 Institut Pasteur, Centre François Jacob, Paris, France; Institut Necker-Enfants-Malades, FRANCE

## Abstract

*Neisseria* adhesin A (NadA) is present on the meningococcal surface and contributes to adhesion to and invasion of human cells. NadA is also one of three recombinant antigens in the recently-approved *Bexsero* vaccine, which protects against serogroup B meningococcus. The amount of NadA on the bacterial surface is of direct relevance in the constant battle of host-pathogen interactions: it influences the ability of the pathogen to engage human cell surface-exposed receptors and, conversely, the bacterial susceptibility to the antibody-mediated immune response. It is therefore important to understand the mechanisms which regulate *nadA* expression levels, which are predominantly controlled by the transcriptional regulator NadR (*Neisseria* adhesin A Regulator) both *in vitro* and *in vivo*. NadR binds the *nadA* promoter and represses gene transcription. In the presence of 4-hydroxyphenylacetate (4-HPA), a catabolite present in human saliva both under physiological conditions and during bacterial infection, the binding of NadR to the *nadA* promoter is attenuated and *nadA* expression is induced. NadR also mediates ligand-dependent regulation of many other meningococcal genes, for example the highly-conserved multiple adhesin family (*maf*) genes, which encode proteins emerging with important roles in host-pathogen interactions, immune evasion and niche adaptation. To gain insights into the regulation of NadR mediated by 4-HPA, we combined structural, biochemical, and mutagenesis studies. In particular, two new crystal structures of ligand-free and ligand-bound NadR revealed (i) the molecular basis of ‘conformational selection’ by which a single molecule of 4-HPA binds and stabilizes dimeric NadR in a conformation unsuitable for DNA-binding, (ii) molecular explanations for the binding specificities of different hydroxyphenylacetate ligands, including 3Cl,4-HPA which is produced during inflammation, (iii) the presence of a leucine residue essential for dimerization and conserved in many MarR family proteins, and (iv) four residues (His7, Ser9, Asn11 and Phe25), which are involved in binding 4-HPA, and were confirmed *in vitro* to have key roles in the regulatory mechanism in bacteria. Overall, this study deepens our molecular understanding of the sophisticated regulatory mechanisms of the expression of *nadA* and other genes governed by NadR, dependent on interactions with niche-specific signal molecules that may play important roles during meningococcal pathogenesis.

## Author Summary

Serogroup B meningococcus (MenB) causes fatal sepsis and invasive meningococcal disease, particularly in young children and adolescents, as highlighted by recent MenB outbreaks in universities of the United States and Canada. The Bexsero vaccine protects against MenB and has recently been approved in > 35 countries worldwide. *Neisseria* adhesin A (NadA) present on the meningococcal surface can mediate binding to human cells and is one of the three MenB vaccine protein antigens. The amount of NadA exposed on the meningococcal surface also influences the antibody-mediated serum bactericidal response measured *in vitro*. A deep understanding of *nadA* expression is therefore important, otherwise the contribution of NadA to vaccine-induced protection against meningococcal meningitis may be underestimated. The abundance of surface-exposed NadA is regulated by the ligand-responsive transcriptional repressor NadR. Here, we present functional, biochemical and high-resolution structural data on NadR. Our studies provide detailed insights into how small molecule ligands, such as hydroxyphenylacetate derivatives, found in relevant host niches, modulate the structure and activity of NadR, by ‘conformational selection’ of inactive forms. These findings shed light on the regulation of NadR, a key MarR-family virulence factor of this important human pathogen.

## Introduction

The ‘Reverse Vaccinology’ approach was pioneered to identify antigens for a protein-based vaccine against serogroup B *Neisseria meningitidis* (MenB), a human pathogen causing potentially-fatal sepsis and invasive meningococcal disease [1]. Indeed, Reverse Vaccinology identified *Neisseria* adhesin A (NadA), a surface-exposed protein involved in epithelial cell invasion and found in ~30% of clinical isolates [2–4]. Recently, we reported the crystal structure of NadA, providing insights into its biological and immunological functions [5]. Recombinant NadA elicits a strong bactericidal immune response and is therefore included in the *Bexsero* vaccine that protects against MenB and which was recently approved in over 35 countries worldwide [6].

Previous studies revealed that *nadA* expression levels are mainly regulated by the *Neisseria* adhesin A Regulator (NadR) [7]. Although additional factors influence *nadA* expression, we focused on its regulation by NadR, the major mediator of NadA phase variable expression [8, 9]. Studies of NadR also have broader implications, since a genome-wide analysis of MenB wild-type and *nadR* knock-out strains revealed that NadR influences the regulation of > 30 genes, including *maf* genes, from the multiple adhesin family [10]. These genes encode a wide variety of proteins connected to many biological processes contributing to bacterial survival, adaptation in the host niche, colonization and invasion [11, 12].

NadR belongs to the MarR (Multiple Antibiotic Resistance Regulator) family, a group of ligand-responsive transcriptional regulators ubiquitous in bacteria and archaea. MarR family proteins can promote bacterial survival in the presence of antibiotics, toxic chemicals, organic solvents or reactive oxygen species [13, 14] and can regulate virulence factor expression [15]. MarR homologues can act either as transcriptional repressors or as activators [16]. Although > 50 MarR family structures are known, a molecular understanding of their ligand-dependent regulatory mechanisms is still limited, often hampered by lack of identification of their ligands and/or DNA targets. A potentially interesting exception comes from the ligand-free and salicylate-bound forms of the *Methanobacterium thermoautotrophicum* protein MTH313 which revealed that two salicylate molecules bind to one MTH313 dimer and induce large conformational changes, apparently sufficient to prevent DNA binding [17]. However, the homologous archeal *Sulfolobus tokodaii* protein ST1710 presented essentially the same structure in ligand-free and salicylate-bound forms, apparently contrasting the mechanism proposed for MTH313 [18]. Despite these apparent differences, MTH313 and ST1710 bind salicylate in approximately the same site, between their dimerization and DNA-binding domains. However, it is unknown whether salicylate is a relevant *in vivo* ligand of either of these two proteins, which share ~20% sequence identity with NadR, rendering unclear the interpretation of these findings in relation to the regulatory mechanisms of NadR or other MarR family proteins [16].

NadR binds the *nadA* promoter and represses gene transcription [9]. NadR binds *nadA* on three different operators (OpI, OpII and OpIII) [10]. The DNA-binding activity of NadR is attenuated *in vitro* upon addition of various hydroxyphenylacetate (HPA) derivatives, including 4-HPA. 4-HPA is a small molecule derived from mammalian aromatic amino acid catabolism and is released in human saliva, where it has been detected at micromolar concentration [19]. In the presence of 4-HPA, NadR is unable to bind the *nadA* promoter and *nadA* gene expression is induced [9, 10]. *In vivo*, the presence of 4-HPA in the host niche of *N*. *meningitidis* serves as an inducer of NadA production, thereby promoting bacterial adhesion to host cells [10]. Further, we recently reported that 3Cl,4-HPA, produced during inflammation, is another inducer of *nadA* expression [20].

Extending our previous studies based on hydrogen-deuterium exchange mass spectrometry (HDX-MS) [21], here we sought to reveal the molecular mechanisms and effects of NadR/HPA interactions via X-ray crystallography, NMR spectroscopy and complementary biochemical and *in vivo* mutagenesis studies. We obtained detailed new insights into ligand specificity, how the ligand allosterically influences the DNA-binding ability of NadR, and the regulation of *nadA* expression, thus also providing a deeper structural understanding of the ligand-responsive MarR super-family. Moreover, these findings are important because the activity of NadR impacts the potential coverage provided by anti-NadA antibodies elicited by the *Bexsero* vaccine and influences host-bacteria interactions that contribute to meningococcal pathogenesis [20].

## Results

### NadR is dimeric and is stabilized by specific hydroxyphenylacetate ligands

Recombinant NadR was produced in *E*. *coli* using an expression construct prepared from *N*. *meningitidis* serogroup B strain MC58. Standard chromatographic techniques were used to obtain a highly purified sample of NadR (see *Materials and Methods*). In analytical size-exclusion high-performance liquid chromatography (SE-HPLC) experiments coupled with multi-angle laser light scattering (MALLS), NadR presented a single species with an absolute molecular mass of 35 kDa (S1 Fig). These data showed that NadR was dimeric in solution, since the theoretical molecular mass of the NadR dimer is 33.73 kDa; and, there was no change in oligomeric state on addition of 4-HPA.

**Fig 1:**
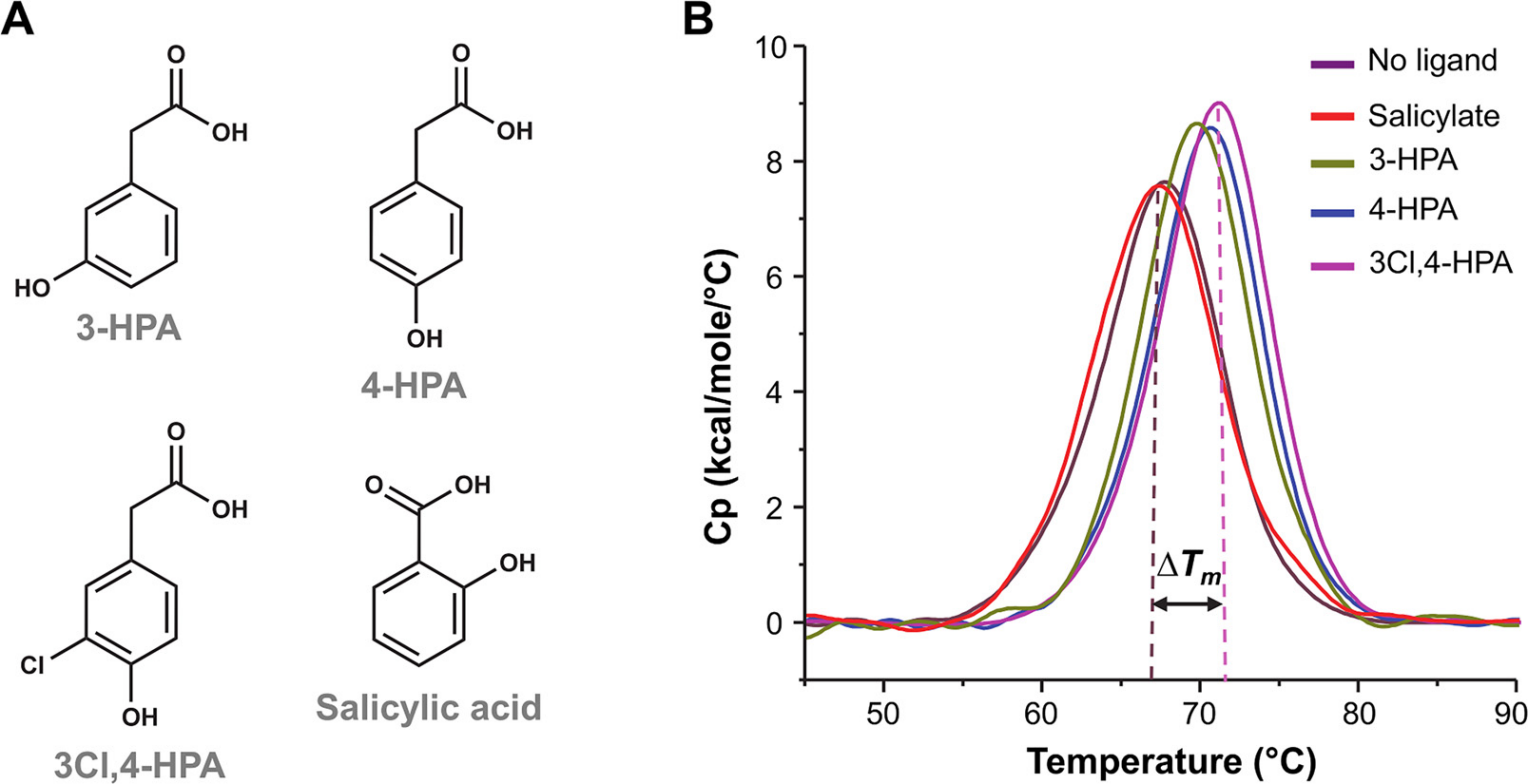
Stability of NadR is increased by small molecule ligands. **(A)** Molecular structures of 3-HPA (MW 152.2), 4-HPA (MW 152.2), 3Cl,4-HPA (MW 186.6) and salicylic acid (MW 160.1). **(B)** DSC profiles, colored as follows: apo-NadR (violet), NadR+salicylate (red), NadR+3-HPA (green), NadR+4-HPA (blue), NadR+3Cl,4-HPA (pink). All DSC profiles are representative of triplicate experiments.

The thermal stability of NadR was examined using differential scanning calorimetry (DSC). Since ligand-binding often increases protein stability, we also investigated the effect of various HPAs (Fig 1A) on the melting temperature (T_m_) of NadR. As a control of specificity, we also tested salicylate, a known ligand of some MarR proteins previously reported to increase the T_m_ of ST1710 and MTH313 [17]. The T_m_ of NadR was 67.4 ± 0.1°C in the absence of ligand, and was unaffected by salicylate. However, an increased thermal stability was induced by 4-HPA and, to a lesser extent, by 3-HPA. Interestingly, NadR displayed the greatest T_m_ increase upon addition of 3Cl,4-HPA (Table 1 and Fig 1B).

**Table 1:** Melting-point (T_m_) and its ligand-induced increase (ΔT_m_) derived from DSC thermostability experiments. Dissociation constants (K_**D**_) of the NadR/ligand interactions from SPR steady-state binding experiments.

Ligand	T_m_ (°C)	ΔT_m_ (°C)	K_D_ (mM)
No ligand	67.4 ± 0.1	n.a.	n.a.
Salicylate	67.5 ± 0.1	0	n.d.
3-HPA	70.0 ± 0.1	2.7	2.7 ± 0.1
4-HPA	70.7 ± 0.1	3.3	1.5 ± 0.1
3Cl,4-HPA	71.3 ± 0.2	3.9	1.1 ± 0.1

n.a.: not applicable; n.d.: not determinable

### NadR displays distinct binding affinities for hydroxyphenylacetate ligands

To further investigate the binding of HPAs to NadR, we used surface plasmon resonance (SPR). The SPR sensorgrams revealed very fast association and dissociation events, typical of small molecule ligands, thus prohibiting a detailed study of binding kinetics. However, steady-state SPR analyses of the NadR-HPA interactions allowed determination of the equilibrium dissociation constants (K_D_) (Table 1 and S2 Fig). The interactions of 4-HPA and 3Cl,4-HPA with NadR exhibited K_D_ values of 1.5 mM and 1.1 mM, respectively. 3-HPA showed a weaker interaction, with a K_D_ of 2.7 mM, while salicylate showed only a very weak response that did not reach saturation, indicating a non-specific interaction with NadR. A ranking of these K_D_ values showed that 3Cl,4-HPA was the tightest binder, and thus matched the ranking of ligand-induced T_m_ increases observed in the DSC experiments. Although these K_D_ values indicate rather weak interactions, they are similar to the values reported previously for the MarR/salicylate interaction (K_D_ ~1 mM) [22] and the MTH313/salicylate interaction (K_D_ 2–3 mM) [17], and approximately 20-fold tighter than the ST1710/salicylate interaction (K_D_ ~20 mM) [18].

### Crystal structures of holo-NadR and apo-NadR

To fully characterize the NadR/HPA interactions, we sought to determine crystal structures of NadR in ligand-bound (holo) and ligand-free (apo) forms. First, we crystallized NadR (a selenomethionine-labelled derivative) in the presence of a 200-fold molar excess of 4-HPA. The structure of the NadR/4-HPA complex was determined at 2.3 Å resolution using a combination of the single-wavelength anomalous dispersion (SAD) and molecular replacement (MR) methods, and was refined to *R*_work_/*R*_free_ values of 20.9/26.0% (Table 2). Despite numerous attempts, we were unable to obtain high-quality crystals of NadR complexed with 3Cl,4-HPA, 3,4-HPA, 3-HPA or DNA targets. However, it was eventually possible to crystallize apo-NadR, and the structure was determined at 2.7 Å resolution by MR methods using the NadR/4-HPA complex as the search model. The apo-NadR structure was refined to *R*_work_/*R*_free_ values of 19.1/26.8% (Table 2).

**Table 2:** Data collection and refinement statistics for NadR structures.

	NadR SeMet + 4-HPA (SAD peak) (PDB code 5aip)	NadR apo-form (PDB code 5aiq)
***Data collection***		
Wavelength (Å)	0.9792	1.0
Beamline	SLS (PXII-X10SA)	SLS (PXII-X10SA)
Resolution range (Å)	39.2–2.3	48.2–2.7
Space group	P 43 21 2	P 43 21 2
Unit cell dimensions (Å)	75.3, 75.3, 91.8	69.4, 69.4, 253.8
Total reflections	291132 (41090)	225521 (35809)
Unique reflections	12320 (1773)	17700 (2780)
Multiplicity	23.6 (23.2)	12.7 (12.8)
Completeness (%)	100.0 (100.00)	99.9 (99.7)
Mean I/sigma(I)	25.5 (9.0)	22.6 (3.8)
Wilson B-factor	23.9	49.1
*R*_sym_ *	10.9 (39.4)	11.4 (77.6)
*R*_meas_ **	11.3	11.8
***Refinement***		
*R*_work_ ^♯^	20.9	21.7
*R*_free_ ^♯♯^	26.0	27.2
*Number of atoms*		
Non-hydrogen atoms	2263	4163
Macromolecules	2207	4144
Ligands	11	0
Water	45	19
Protein residues	275	521
RMS(bonds)	0.008	0.003
RMS(angles)	1.09	0.823
*Ramachandran (%)* ^§^		
Favored	100	98.4
Outliers	0	0
Clash score	5.0	3.9
*Average B-factor*		
Macromolecules	34.8	53.3
Ligands	32.9	-
Solvent	37.3 (H_2_O)	29.0 (H_2_O)

Statistics for the highest-resolution shell are shown in parentheses.

*R_sym_ = Σ_hkl_ Σ_i_ |I_i_(hkl)—<I(hkl)>| / Σ_hkl_ Σ_i_ I_i_(hkl)

** *R*_meas_ = redundancy-independent (multiplicity-weighted) *R*_merge_ as reported from AIMLESS [43].

^**♯**^ *R*_work_ = Σ||F_(obs)_|- |F_(calc)_||/Σ|F_(obs)_|

^**♯♯**^ *R*_free_ = as for *R*_work_, calculated for 5.0% of the total reflections, chosen at random, and omitted from refinement.

^**§**^ Values obtained using Molprobity [41].

The asymmetric unit of the NadR/4-HPA crystals (holo-NadR) contained one NadR homodimer, while the apo-NadR crystals contained two homodimers. In the apo-NadR crystals, the two homodimers were related by a rotation of ~90°; the observed association of the two dimers was presumably merely an effect of crystal packing, since the interface between the two homodimers is small (< 550 Å^2^ of buried surface area), and is not predicted to be physiologically relevant by the PISA software [23]. Moreover, our SE-HPLC/MALLS analyses (see above) revealed that in solution NadR is dimeric, and previous studies using native mass spectrometry (MS) revealed dimers, not tetramers [21].

**Fig 2:**
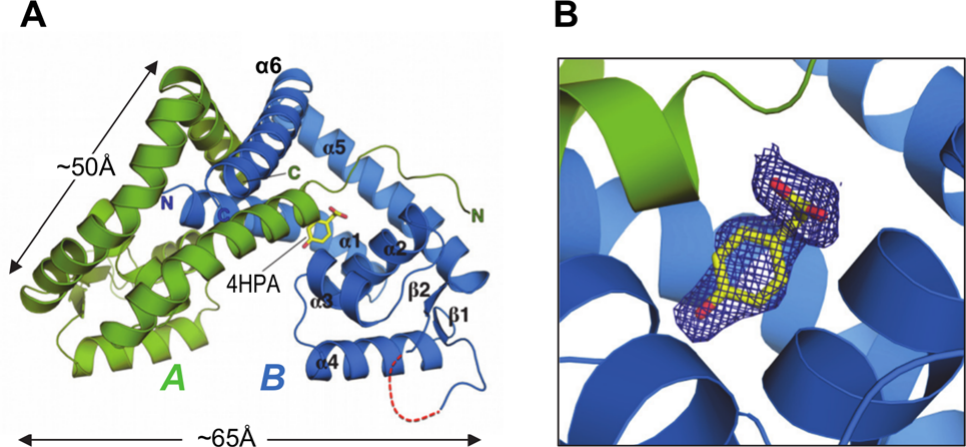
The crystal structure of NadR in complex with 4-HPA. **(A)** The holo-NadR homodimer is depicted in green and blue for chains A and B respectively, while yellow sticks depict the 4-HPA ligand (labelled). For simplicity, secondary structure elements are labelled for chain B only. Red dashes show hypothetical positions of chain B residues 88–90 that were not modeled due to lack of electron density. **(B)** A zoom into the pocket occupied by 4-HPA shows that the ligand contacts both chains A and B; blue mesh shows electron density around 4-HPA calculated from a composite omit map (omitting 4-HPA), using *phenix* [38]. The map is contoured at 1σ and the figure was prepared with a density mesh carve factor of 1.7, using Pymol (www.pymol.org).

The NadR homodimer bound to 4-HPA has a dimerization interface mostly involving the top of its ‘triangular’ form, while the two DNA-binding domains are located at the base (Fig 2A). High-quality electron density maps allowed clear identification of the bound ligand, 4-HPA (Fig 2B). The overall structure of NadR shows dimensions of ~50 × 65 × 50 Å and a large homodimer interface that buries a total surface area of ~ 4800 Å^2^. Each NadR monomer consists of six α-helices and two short β-strands, with helices α1, α5, and α6 forming the dimer interface. Helices α3 and α4 form a helix-turn-helix motif, followed by the “wing motif” comprised of two short antiparallel β-strands (β1-β2) linked by a relatively long and flexible loop. Interestingly, in the α4-β2 region, the stretch of residues from R64-R91 presents seven positively-charged side chains, all available for potential interactions with DNA. Together, these structural elements constitute the winged helix-turn-helix (wHTH) DNA-binding domain and, together with the dimeric organization, are the hallmarks of MarR family structures [16].

### A single conserved leucine residue (L130) is crucial for dimerization

**Fig 3:**
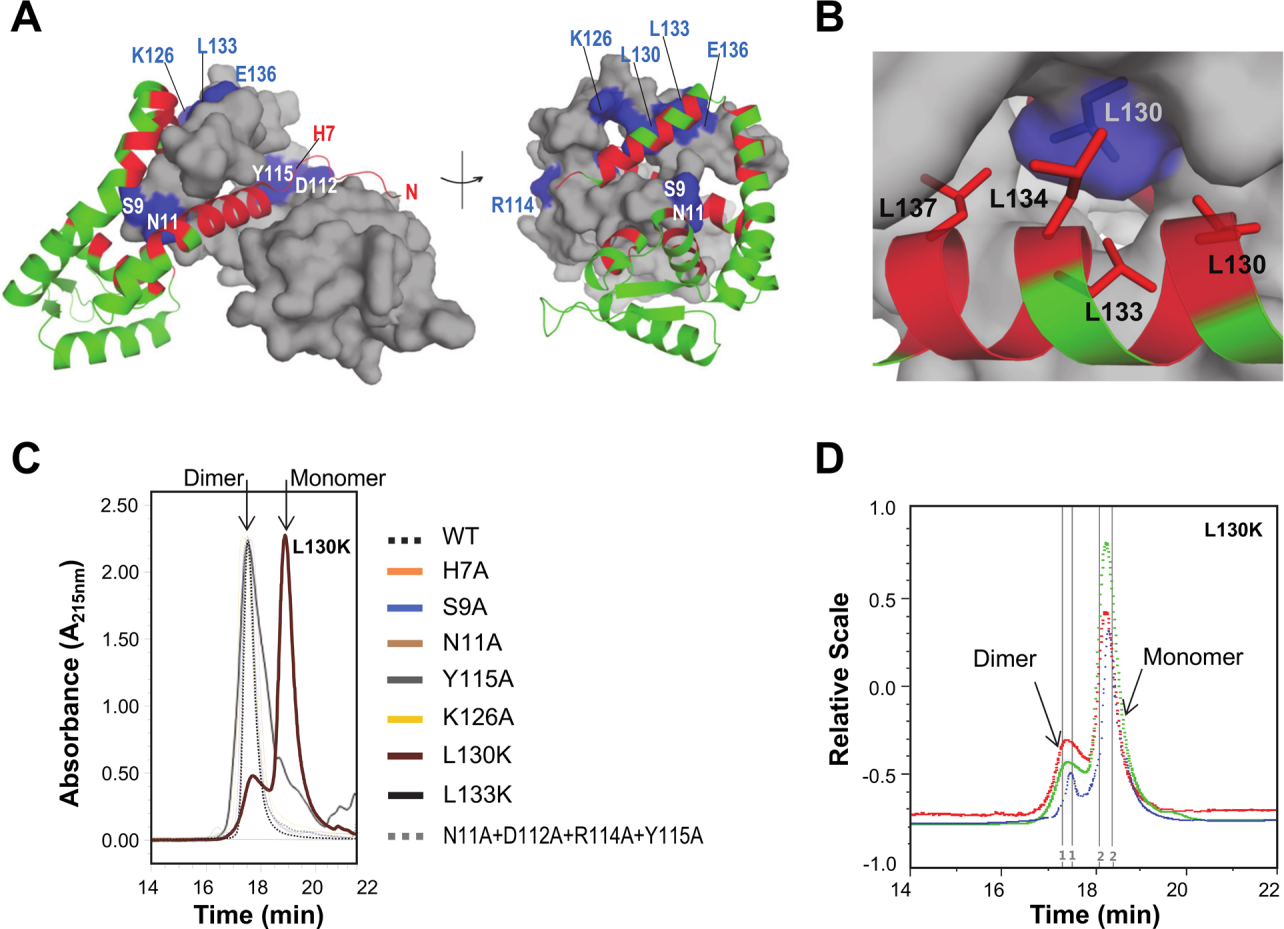
Analysis of the NadR dimer interface. **(A)** Both orientations show chain A, green backbone ribbon, colored red to highlight all locations involved in dimerization; namely, inter-chain salt bridges or hydrogen bonds involving Q4, S5, K6, H7, S9, I10, N11, I15, Q16, R18, D36, R43, A46, Q59, C61, Y104, D112, R114, Y115, D116, E119, K126, E136, E141, N145, and the hydrophobic packing interactions involving I10, I12, L14, I15, R18, Y115, I118, L130, L133, L134 and L137. Chain B, grey surface, is marked blue to highlight residues probed by site-directed mutagenesis (E136 only makes a salt bridge with K126, therefore it was sufficient to make the K126A mutation to assess the importance of this ionic interaction; the H7 position is labelled for monomer A, since electron density was lacking for monomer B). **(B)** A zoom into the environment of helix α6 to show how residue L130 chain B (blue side chain) is a focus of hydrophobic packing interactions with L130, L133, L134 and L137 of chain A (red side chains). **(C)** SE-HPLC analyses of all mutant forms of NadR are compared with the wild-type (WT) protein. The WT and most of the mutants show a single elution peak with an absorbance maximum at 17.5 min. Only the mutation L130K has a noteworthy effect on the oligomeric state, inducing a second peak with a longer retention time and a second peak maximum at 18.6 min. To a much lesser extent, the L133K mutation also appears to induce a ‘shoulder’ to the main peak, suggesting very weak ability to disrupt the dimer. **(D)** SE-HPLC/MALLS analyses of the L130K mutant, shows 20% dimer and 80% monomer. The curves plotted correspond to Absorbance Units (mAU) at 280nm wavelength (green), light scattering (red), and refractive index (blue).

The NadR dimer interface is formed by at least 32 residues, which establish numerous inter-chain salt bridges or hydrogen bonds, and many hydrophobic packing interactions (Fig 3A and 3B). To determine which residues were most important for dimerization, we studied the interface *in silico* and identified several residues as potential mediators of key stabilizing interactions. Using site-directed mutagenesis, a panel of eight mutant NadR proteins was prepared (including mutations H7A, S9A, N11A, D112A, R114A, Y115A, K126A, L130K and L133K), sufficient to explore the entire dimer interface. Each mutant NadR protein was purified, and then its oligomeric state was examined by analytical SE-HPLC. Almost all the mutants showed the same elution profile as the wild-type (WT) NadR protein. Only the L130K mutation induced a notable change in the oligomeric state of NadR (Fig 3C). Further, in SE-MALLS analyses, the L130K mutant displayed two distinct species in solution, approximately 80% being monomeric (a 19 kDa species), and only 20% retaining the typical native dimeric state (a 35 kDa species) (Fig 3D), demonstrating that Leu130 is crucial for stable dimerization. It is notable that L130 is usually present as Leu, or an alternative bulky hydrophobic amino acid (e.g. Phe, Val), in many MarR family proteins, suggesting a conserved role in stabilizing the dimer interface. In contrast, most of the other residues identified in the NadR dimer interface were poorly conserved in the MarR family.

### The holo-NadR structure presents only one occupied ligand-binding pocket

The NadR/4-HPA structure revealed the ligand-binding site nestled between the dimerization and DNA-binding domains (Fig 2). The ligand showed a different position and orientation compared to salicylate complexed with MTH313 and ST1710 [17, 18] (see Discussion). The binding pocket was almost entirely filled by 4-HPA and one water molecule, although there also remained a small tunnel 2-4Å in diameter and 5-6Å long leading from the pocket (proximal to the 4-hydroxyl position) to the protein surface. The tunnel was lined with rather hydrophobic amino acids, and did not contain water molecules. Unexpectedly, only one monomer of the holo-NadR homodimer contained 4-HPA in the binding pocket, whereas the corresponding pocket of the other monomer was unoccupied by ligand, despite the large excess of 4-HPA used in the crystallization conditions.

**Fig 4:**
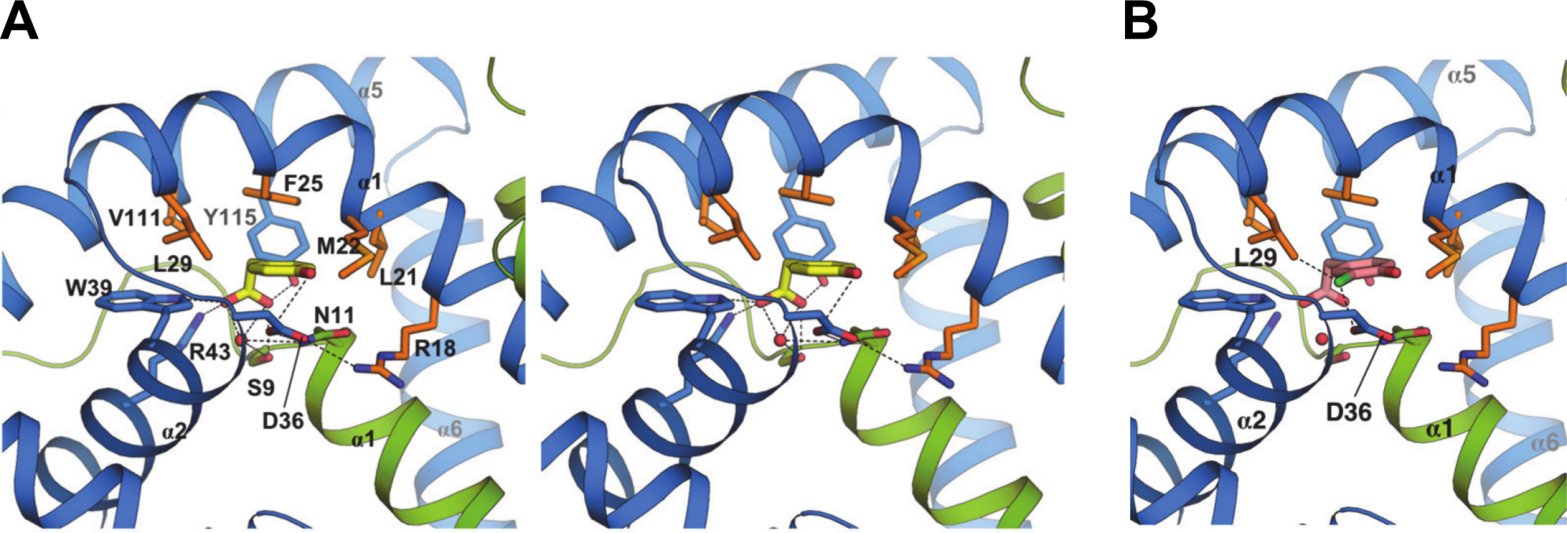
Atomic details of NadR/HPA interactions. **A)** A stereo-view zoom into the binding pocket showing side chain sticks for all interactions between NadR and 4-HPA. Green and blue ribbons depict NadR chains A and B, respectively. 4-HPA is shown in yellow sticks, with oxygen atoms in red. A water molecule is shown by the red sphere. H-bonds up to 3.6Å are shown as dashed lines. The entire set of residues making H-bonds or non-bonded contacts with 4-HPA is as follows: SerA9, AsnA11, LeuB21, MetB22, PheB25, LeuB29, AspB36, TrpB39, ArgB43, ValB111 and TyrB115 (automated analysis performed using PDBsum [44] and verified manually). Residues AsnA11 and ArgB18 likely make indirect yet local contributions to ligand binding, mainly by stabilizing the position of AspB36. Bond distances for interacting polar atoms are provided in Table 3. Side chains mediating hydrophobic interactions are shown in orange. **(B)** A model was prepared to visualize putative interactions of 3Cl,4-HPA (pink) with NadR, revealing the potential for additional contacts (dashed lines) of the chloro moiety (green stick) with LeuB29 and AspB36.

Inspection of the protein-ligand interaction network revealed no bonds from NadR backbone groups to the ligand, but several key side chain mediated hydrogen (H)-bonds and ionic interactions, most notably between the carboxylate group of 4-HPA and Ser9 of chain A (SerA9), and chain B residues TrpB39, ArgB43 and TyrB115 (Fig 4A). At the other ‘end’ of the ligand, the 4-hydroxyl group was proximal to AspB36, with which it may establish an H-bond (see bond distances in Table 3). The water molecule observed in the pocket was bound by the carboxylate group and the side chains of SerA9 and AsnA11.

**Table 3:** List of 4-HPA atoms bound to NadR via ionic interactions and/or H-bonds.

4-HPA atom	NadR residue/atom	Distance (Å)
O2	TrpB39/NE1	2.83
O2	ArgB43/NH1	2.76
O1	ArgB43/NH1	3.84
O1	SerA9/OG	2.75
O1	TyrB115/OH	2.50
O2	Water (*Ser9/Asn11)	2.88
OH	AspB36/OD1/OD2	3.6/3.7

* Bond distance between the ligand carboxylate group and the water molecule, which in turn makes H-bond to the SerA9 and AsnA11 side chains.

In addition to the H-bonds involving the carboxylate and hydroxyl groups of 4-HPA, binding of the phenyl moiety appeared to be stabilized by several van der Waals’ contacts, particularly those involving the hydrophobic side chain atoms of LeuB21, MetB22, PheB25, LeuB29 and ValB111 (Fig 4A). Notably, the phenyl ring of PheB25 was positioned parallel to the phenyl ring of 4-HPA, potentially forming π-π parallel-displaced stacking interactions. Consequently, residues in the 4-HPA binding pocket are mostly contributed by NadR chain B, and effectively created a polar ‘floor’ and a hydrophobic ‘ceiling’, which house the ligand. Collectively, this mixed network of polar and hydrophobic interactions endows NadR with a strong recognition pattern for HPAs, with additional medium-range interactions potentially established with the hydroxyl group at the 4-position.

### Structure-activity relationships: molecular basis of enhanced stabilization by 3Cl,4-HPA

We modelled the binding of other HPAs by *in silico* superposition onto 4-HPA in the holo-NadR structure, and thereby obtained molecular explanations for the binding specificities of diverse ligands. For example, similar to 4-HPA, the binding of 3Cl,4-HPA could involve multiple bonds towards the carboxylate group of the ligand and some to the 4-hydroxyl group. Additionally, the side chains of LeuB29 and AspB36 would be only 2.6–3.5 Å from the chlorine atom, thus providing van der Waals’ interactions or H-bonds to generate the additional binding affinity observed for 3Cl,4-HPA (Fig 4B). The presence of a single hydroxyl group at position 2, as in 2-HPA, rather than at position 4, would eliminate the possibility of favorable interactions with Asp*B*36, resulting in the lack of NadR regulation by 2-HPA described previously [20]. Finally, salicylate is presumably unable to specifically bind NadR due to the 2-hydroxyl substitution and the shorter aliphatic chain connecting its carboxylate group (Fig 1A): the compound simply seems too small to simultaneously establish the network of beneficial bonds observed in the NadR/HPA interactions.

### Analysis of the pockets reveals the molecular basis for asymmetric binding and stoichiometry

We attempted to investigate further the binding stoichiometry using solution-based techniques. However, studies based on tryptophan fluorescence were confounded by the fluorescence of the HPA ligands, and isothermal titration calorimetry (ITC) was unfeasible due to the need for very high concentrations of NadR in the ITC chamber (due to the relatively low affinity), which exceeded the solubility limits of the protein. However, it was possible to calculate the binding stoichiometry of the NadR-HPA interactions using an SPR-based approach. In SPR, the signal measured is proportional to the total molecular mass proximal to the sensor surface; consequently, if the molecular weights of the interactors are known, then the stoichiometry of the resulting complex can be determined [24]. This approach relies on the assumption that the captured protein (‘the ligand’, according to SPR conventions) is 100% active and freely-accessible to potential interactors (‘the analytes’). This assumption is likely valid for this pair of interactors, for two main reasons. Firstly, NadR is expected to be covalently immobilized on the sensor chip as a dimer in random orientations, since it is a stable dimer in solution and has sixteen lysines well-distributed around its surface, all able to act as potential sites for amine coupling to the chip, and none of which are close to the ligand-binding pocket. Secondly, the HPA analytes are all very small (MW 150–170, Fig 1A) and therefore are expected to be able to diffuse readily into all potential binding sites, irrespective of the random orientations of the immobilized NadR dimers on the chip.The stoichiometry of the NadR-HPA interactions was determined using Eq 1 (see Materials and Methods), and revealed stoichiometries of 1.13 for 4-HPA, 1.02 for 3-HPA, and 1.21 for 3Cl,4-HPA, strongly suggesting that one NadR dimer bound to 1 HPA analyte molecule.

**Fig 5:**
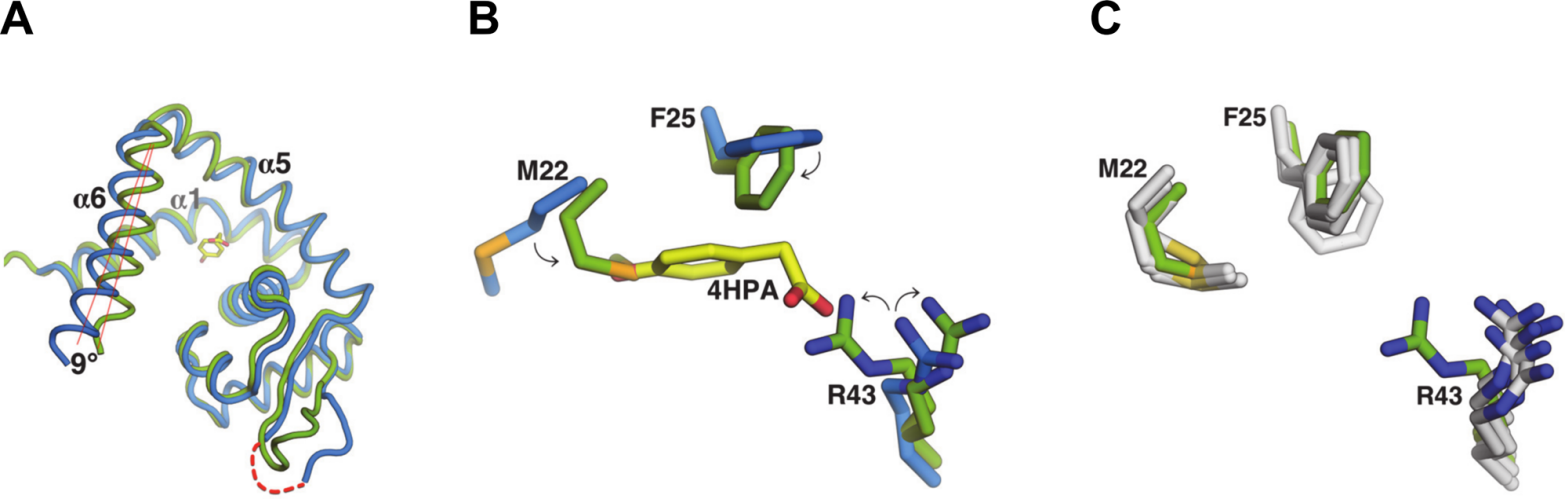
Structural differences of NadR in ligand-bound or free forms. **(A)** Aligned monomers of holo-NadR (chain A: green; chain B: blue), reveal major overall differences by the shift of helix α6. **(B)** Comparison of the two binding pockets in holo-NadR shows that in the ligand-free monomer A (green) residues Met22, Phe25 and Arg43 adopt ‘inward’ positions (highlighted by arrows) compared to the ligand-occupied pocket (blue residues); these ‘inward’ conformations appear unfavorable for binding of 4-HPA due to clashes with the 4-hydroxyl group, the phenyl ring and the carboxylate group, respectively. In these crystals, the ArgA43 side chain showed two alternate conformations, modelled with 50% occupancy in each state, as indicated by the two ‘mirrored’ arrows. The inner conformer is the one that would display major clashes if 4-HPA were present. **(C)** Comparison of the empty pocket from holo-NadR (green residues) with the four empty pockets of apo-NadR (grey residues), shows that in the absence of 4-HPA the Arg43 side chain is always observed in the ‘outward’ conformation.

The crystallographic data, supported by the SPR studies of binding stoichiometry, revealed the lack of a second 4-HPA molecule in the homodimer, suggesting negative co-operativity, a phenomenon previously described for the MTH313/salicylate interaction [17] and for other MarR family proteins [16]. To explore the molecular basis of asymmetry in holo-NadR, we superposed its ligand-free monomer (chain A) onto the ligand-occupied monomer (chain B). Overall, the superposition revealed a high degree of structural similarity (Cα root mean square deviation (rmsd) of 1.5Å), though on closer inspection a rotational difference of ~9 degrees along the long axis of helix α6 was observed, suggesting that 4-HPA induced a slight conformational change (Fig 5A). However, since residues of helix α6 were not directly involved in ligand binding, an explanation for the lack of 4-HPA in monomer A did not emerge by analyzing only these backbone atom positions, suggesting that a more complex series of allosteric events may occur. Indeed, we noted interesting differences in the side chains of Met22, Phe25 and Arg43, which in monomer B are used to contact the ligand while in monomer A they partially occupied the pocket and collectively reduced its volume significantly. Specifically, upon analysis with the CASTp software [25], the pocket in chain B containing the 4-HPA exhibited a total volume of approximately 370 Å^3^, while the pocket in chain A was occupied by these three side chains that adopted ‘inward’ positions and thereby divided the space into a few much smaller pockets, each with volume < 50 Å^3^, evidently rendering chain A unfavorable for ligand binding. Most notably, atomic clashes between the ligand and the side chains of MetA22, PheA25 and ArgA43 would occur if 4-HPA were present in the monomer A pocket (Fig 5B). Subsequently, analyses of the pockets in apo-NadR revealed that in the absence of ligand the long Arg43 side chain was always in the open ‘outward’ position compatible with binding to the 4-HPA carboxylate group. In contrast, the apo-form Met22 and Phe25 residues were still encroaching the spaces of the 4-hydroxyl group and the phenyl ring of the ligand, respectively (Fig 5C). The ‘outward’ position of Arg43 generated an open apo-form pocket with volume approximately 380Å^3^. Taken together, these observations suggest that Arg43 is a major determinant of ligand binding, and that its ‘inward’ position inhibits the binding of 4-HPA to the empty pocket of holo-NadR.

**Fig 6:**
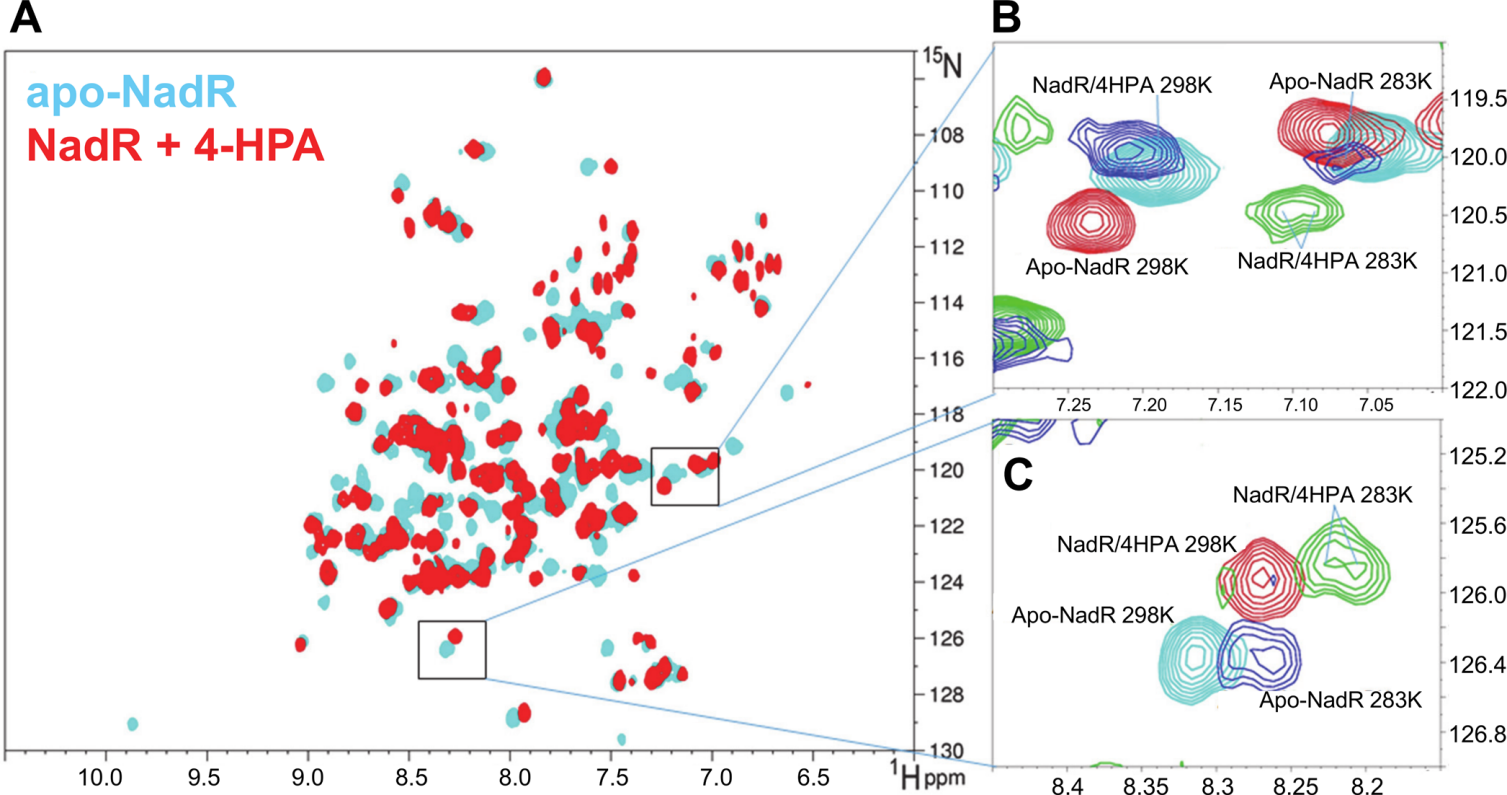
NMR spectra of NadR in the presence and absence of 4-HPA. **(A)** Superposition of two ^1^H-^15^N TROSY-HSQC spectra recorded at 25°C on apo-NadR (cyan) and on NadR in the presence of 4-HPA (red). **(B,C)** Overlay of selected regions of the ^1^H-^15^N TROSY-HSQC spectra acquired at 25°C of apo-NadR (cyan) and NadR/4-HPA (red) superimposed with the spectra acquired at 10°C of apo-NadR (blue) and NadR/4-HPA (green). The spectra acquired at 10°C are excluded from panel A for simplicity.

Finally, we applied ^15^N heteronuclear solution NMR spectroscopy to examine the interaction of 4-HPA with apo NadR. We collected NMR spectra on NadR in the presence and absence of 4-HPA (see Materials and Methods). The ^1^H-^15^N TROSY-HSQC spectrum of apo-NadR, acquired at 25°C, displayed approximately 140 distinct peaks (Fig 6A), most of which correspond to backbone amide N-H groups. The broad spectral dispersion and the number of peaks observed, which is close to the number of expected backbone amide N-H groups for this polypeptide, confirmed that apo-NadR is well-folded under these conditions and exhibits one conformation appreciable on the NMR timescale, i.e. in the NMR experiments at 25°C, two or more distinct conformations of apo-NadR monomers were not readily apparent. Upon the addition of 4-HPA, over 45 peaks showed chemical shift perturbations, i.e. changed position in the spectrum or disappeared, while the remaining peaks remained unchanged. This observation showed that 4-HPA was able to bind NadR and induce notable changes in specific regions of the protein.

However, in the presence of 4-HPA, the ^1^H-^15^N TROSY-HSQC spectrum of NadR displayed approximately 140 peaks, as for apo-NadR, i.e. two distinct stable conformations (that might have potentially revealed the molecular asymmetry observed crystallographically) were not notable. Considering the small size, fast diffusion and relatively low binding affinity of 4-HPA, it would not be surprising if the ligand associates and dissociates rapidly on the NMR time scale, resulting in only one set of peaks whose chemical shifts represent the average environment of the bound and unbound states. Interestingly, by cooling the samples to 10°C, we observed that a number of those peaks strongly affected by 4-HPA (and therefore likely to be in the ligand-binding site) demonstrated evidence of peak splitting, i.e. a tendency to become two distinct peaks rather than one single peak (Fig 6B and 6C). These doubled peaks may therefore reveal that the cooler temperature partially trapped the existence in solution of two distinct states, in presence or absence of 4-HPA, with minor conformational differences occurring at least in proximity to the binding pocket. Although more comprehensive NMR experiments and full chemical shift assignment of the spectra would be required to precisely define this multi-state behavior, the NMR data clearly demonstrate that NadR exhibits conformational flexibility which is modulated by 4-HPA in solution.

### Apo-NadR structures reveal intrinsic conformational flexibility

**Fig 7:**
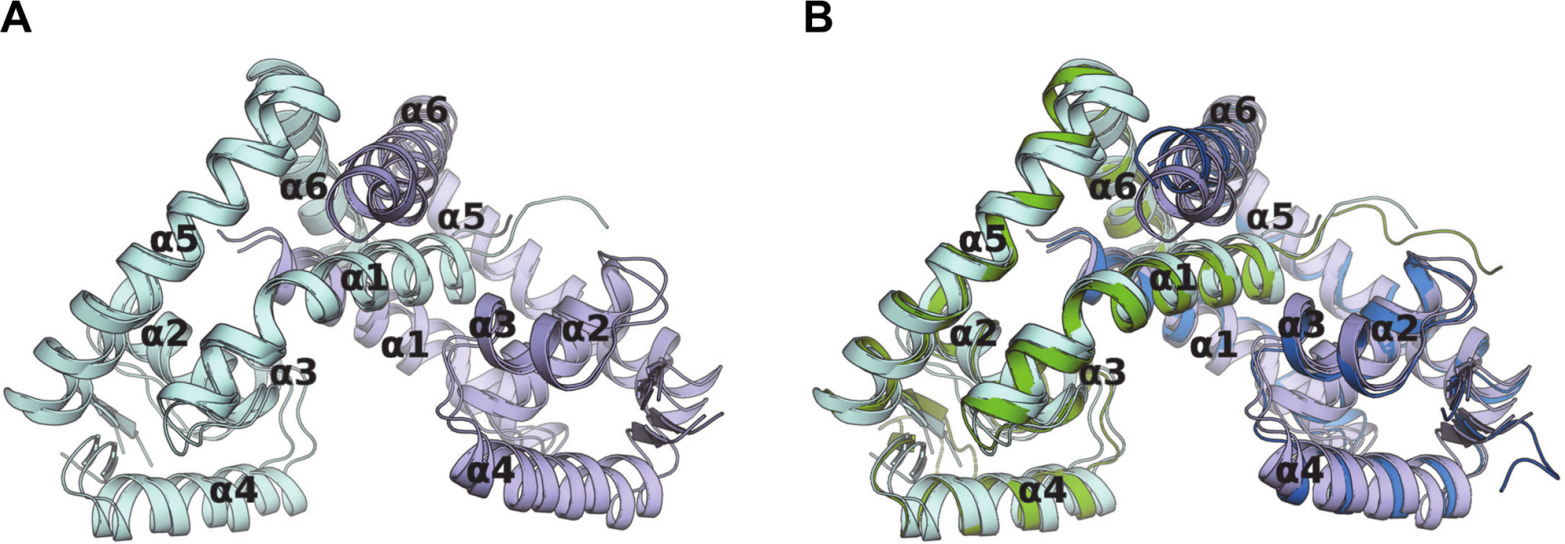
Overall apo- and holo-NadR structures are similar. **(A)** Pairwise alignment of the two distinct apo-NadR homodimers (AB and CD) present in the apo-NadR crystals. **(B)** Alignment of the holo-NadR homodimer (green and blue chains) onto the apo-NadR homodimers. Here, larger differences are observed in the α6 helices (top).

The apo-NadR crystal structure contained two homodimers in the asymmetric unit (chains A+B and chains C+D). Upon overall structural superposition, these dimers revealed a few minor differences in the α6 helix (a major component of the dimer interface) and the helices α4-α5 (the DNA binding region), and an rmsd of 1.55Å (Fig 7A). Similarly, the entire holo-homodimer could be closely superposed onto each of the apo-homodimers, showing rmsd values of 1.29Å and 1.31Å, and with more notable differences in the α6 helix positions (Fig 7B). The slightly larger rmsd between the two apo-homodimers, rather than between apo- and holo-homodimers, further indicate that apo-NadR possesses a notable degree of intrinsic conformational flexibility.

### 4-HPA stabilizes concerted conformational changes in NadR that prevent DNA-binding

**Fig 8:**
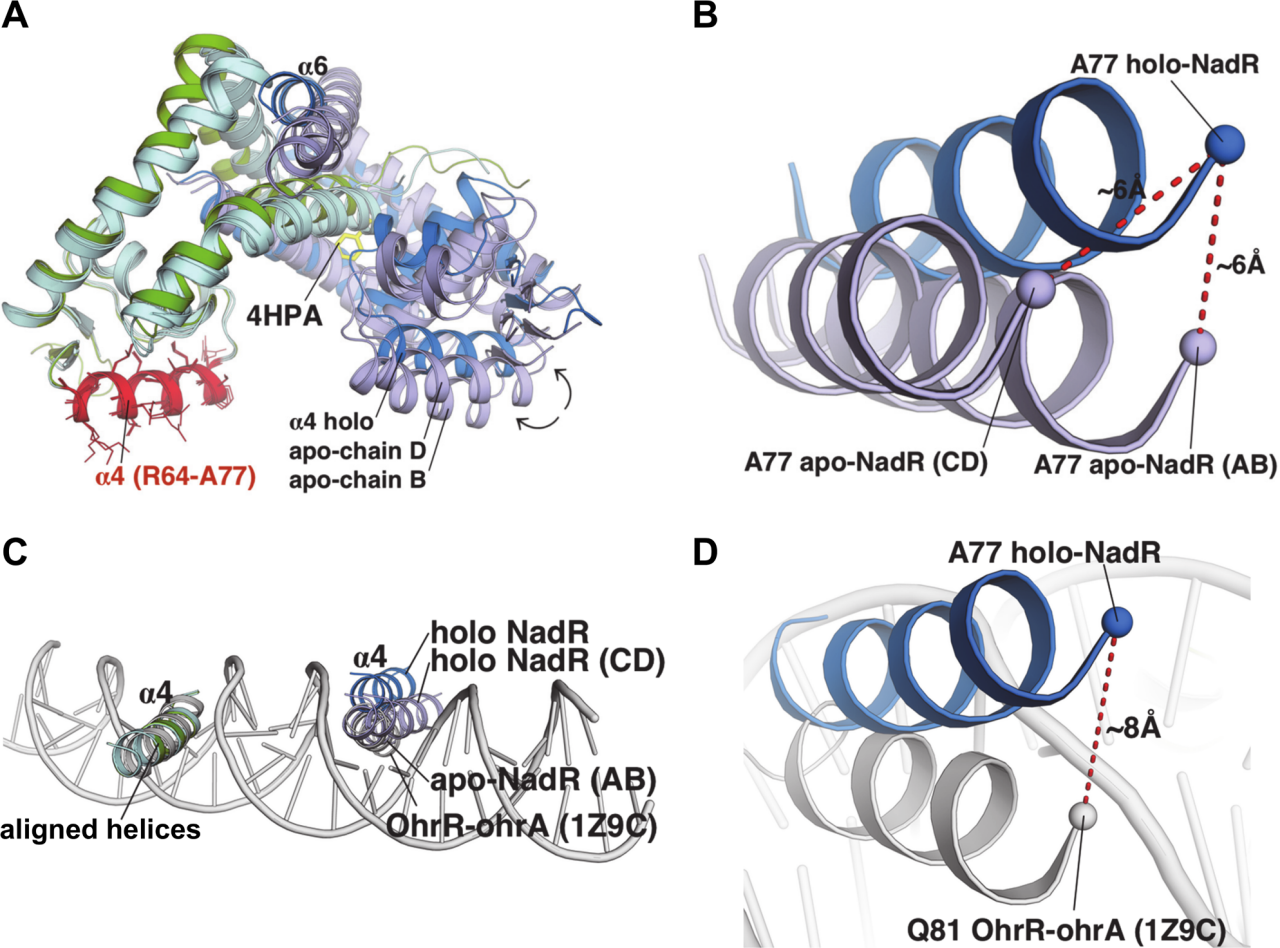
Structural comparisons of NadR and modelling of interactions with DNA. **(A)** The holo-homodimer structure is shown as green and blue cartoons, for chain A and B, respectively, while the two homodimers of apo-NadR are both cyan and pale blue for chains A/C and B/D, respectively. The three homodimers (chains AB holo, AB apo, and CD apo) were overlaid by structural alignment exclusively of all heavy atoms in residues R64-A77 (shown in red, with side chain sticks) of chains A holo, A apo, and C apo, belonging to helix α4 (left). The α4 helices aligned closely, Cα rmsd 0.2Å for 14 residues. **(B)** The relative positions of the α4 helices of the 4-HPA-bound holo homodimer chain B (blue), and of apo homodimers AB and CD (showing chains B and D) in pale blue. Dashes indicate the Ala77 Cα atoms, in the most highly shifted region of the ‘non-fixed’ α4 helix. **(C)** The double-stranded DNA molecule (grey cartoon) from the OhrR-*ohrA* complex is shown after superposition with NadR, to highlight the expected positions of the NadR α4 helices in the DNA major grooves. The proteins share ~30% amino acid sequence identity. For clarity, only the α4 helices are shown in panels (B) and (C). **(D)** Upon comparison with the experimentally-determined OhrR:*ohrA* structure (grey), the α4 helix of holo-NadR (blue) is shifted ~8Å out of the major groove.

To further investigate the conformational rearrangements of NadR, we performed local structural alignments using only a subset of residues in the DNA-binding helix (α4). By selecting and aligning residues Arg64-Ala77 of one α4 helix per dimer, superposition of the holo-homodimer onto the two apo-homodimers revealed differences in the monomer conformations of each structure. While one monomer from each structure was closely superimposable (Fig 8A, left side), the second monomer displayed quite large differences (Fig 8A, right side). Most notably, the position of the DNA-binding helix α4 shifted by as much as 6 Å (Fig 8B). Accordingly, helix α4 was also found to be one of the most dynamic regions in previous HDX-MS analyses of apo-NadR in solution [21].

However, structural comparisons revealed that the shift of holo-NadR helix α4 induced by the presence of 4-HPA was also accompanied by several changes at the holo dimer interface, while such extensive structural differences were not observed in the apo dimer interfaces, particularly notable when comparing the α6 helices (S3 Fig). In summary, compared to ligand-stabilized holo-NadR, apo-NadR displayed an intrinsic flexibility focused in the DNA-binding region. This was also evident in the greater disorder (i.e. less well-defined electron density) in the β1-β2 loops of the apo dimers (density for 16 residues per dimer was missing) compared to the holo dimer (density for only 3 residues was missing).

In holo-NadR, the distance separating the two DNA-binding α4 helices was 32 Å, while in apo-NadR it was 29 Å for homodimer AB, and 34 Å for homodimer CD (Fig 8C). Thus, the apo-homodimer AB presented the DNA-binding helices in a conformation similar to that observed in the protein:DNA complex of OhrR:*ohrA* from *Bacillus subtilis* [26] (Fig 8C). Interestingly, OhrR contacts *ohrA* across 22 base pairs (bp), and similarly the main NadR target sites identified in the *nadA* promoter (the operators Op I and Op II) both span 22 bp [9, 10]. Pairwise superpositions showed that the NadR apo-homodimer AB was the most similar to OhrR (rmsd 2.6 Å), while the holo-homodimer was the most divergent (rmsd 3.3 Å) (Fig 8C). Assuming the same DNA-binding mechanism is used by OhrR and NadR, the apo-homodimer AB seems ideally pre-configured for DNA binding, while 4-HPA appeared to stabilize holo-NadR in a conformation poorly suited for DNA binding. Specifically, in addition to the different inter-helical translational distances, the α4 helices in the holo-NadR homodimer were also reoriented, resulting in movement of α4 out of the major groove, by up to 8Å, and presumably preventing efficient DNA binding in the presence of 4-HPA (Fig 8D). When aligned with OhrR, the apo-homodimer CD presented yet another different intermediate conformation (rmsd 2.9Å), apparently not ideally pre-configured for DNA binding, but which in solution can presumably readily adopt the AB conformation due to the intrinsic flexibility described above.

### NadR residues His7, Ser9, Asn11 and Phe25 are essential for regulation of NadA expression *in vivo*

**Fig 9:**
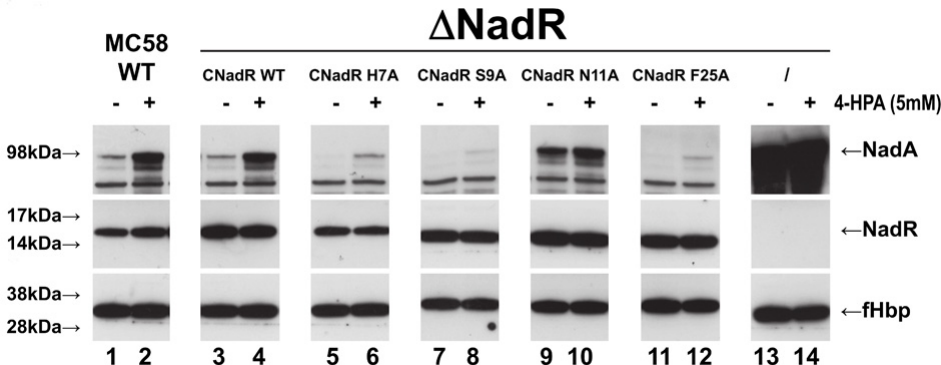
Structure-based point mutations shed light on ligand-induced regulation of NadR. Western blot analyses of wild-type (WT) strain (lanes 1–2) or isogenic *nadR* knockout strains (ΔNadR) complemented to express the indicated NadR WT or mutant proteins (lanes 3–12) or not complemented (lanes 13–14), grown in the presence (even lanes) or absence (odd lanes) of 5mM 4-HPA, showing NadA and NadR expression. Complementation of ΔNadR with WT NadR enables induction of *nadA* expression by 4-HPA. The H7A, S9A and F25A mutants efficiently repress *nadA* expression but are less ligand-responsive than WT NadR. The N11A mutant does not efficiently repress *nadA* expression either in presence or absence of 4-HPA. (The protein abundance levels of the meningococcal factor H binding protein (fHbp) were used as a gel loading control).

While previous studies had correctly suggested the involvement of several NadR residues in ligand binding [21], the crystal structures presented here revealed additional residues with previously unknown roles in dimerization and/or binding to 4-HPA. To explore the functional involvement of these residues, we characterized the behavior of four new NadR mutants (H7A, S9A, N11A and F25A) in an *in vivo* assay using the previously described MC58-Δ1843 *nadR*-null mutant strain [9], which was complemented either by wild-type *nadR* or by the *nadR* mutants. NadA protein abundance levels were assessed by Western blotting to evaluate the ability of the NadR mutants to repress the *nadA* promoter, in the presence or absence of 4-HPA. The *nadR* H7A, S9A and F25A complemented strains showed hyper-repression of *nadA* expression *in vivo*, i.e. these mutants repressed *nadA* more efficiently than the NadR WT protein, either in the presence or absence of 4-HPA, while complementation with wild-type *nadR* resulted in high production of NadA only in the presence of 4-HPA (Fig 9). Interestingly, and on the contrary, the *nadR* N11A complemented strain showed hypo-repression (i.e. exhibited high expression of *nadA* both in absence and presence of 4-HPA). This mutagenesis data revealed that NadR residues His7, Ser9, Asn11 and Phe25 play key roles in the ligand-mediated regulation of NadR; they are each involved in the controlled de-repression of the *nadA* promoter and synthesis of NadA in response to 4-HPA *in vivo*.

## Discussion

NadA is a surface-exposed meningococcal protein contributing to pathogenesis, and is one of three main antigens present in the vaccine *Bexsero* [6]. A detailed understanding of the *in vitro* repression of *nadA* expression by the transcriptional regulator NadR is important, both because it is a relevant disease-related model of how small-molecule ligands can regulate MarR family proteins and thereby impact bacterial virulence, and because *nadA* expression levels are linked to the prediction of vaccine coverage [20] [27]. The repressive activity of NadR can be relieved by hydroxyphenylacetate (HPA) ligands [20], and HDX-MS studies previously indicated that 4-HPA stabilizes dimeric NadR in a configuration incompatible with DNA binding [21]. Despite these and other studies [16], the molecular mechanisms by which ligands regulate MarR family proteins are relatively poorly understood and likely differ depending on the specific ligand. Given the importance of NadR-mediated regulation of NadA levels in the contexts of meningococcal pathogenesis, we sought to characterize NadR, and its interaction with ligands, at atomic resolution.

Firstly, we confirmed that NadR is dimeric in solution and demonstrated that it retains its dimeric state in the presence of 4-HPA, indicating that induction of a monomeric status is not the manner by which 4-HPA regulates NadR. These observations were in agreement with (i) a previous study of NadR performed using SEC and mass spectrometry [21], and (ii) crystallographic studies showing that several MarR homologues are dimeric [16]. We also used structure-guided site-directed mutagenesis to identify an important conserved residue, Leu130, which stabilizes the NadR dimer interface, knowledge of which may also inform future studies to explore the regulatory mechanisms of other MarR family proteins. Secondly, we assessed the thermal stability and unfolding of NadR in the presence or absence of ligands. All DSC profiles showed a single peak, suggesting that a single unfolding event simultaneously disrupted the dimer and the monomer. HPA ligands specifically increased the stability of NadR. The largest effects were induced by the naturally-occurring compounds 4-HPA and 3Cl,4-HPA, which, in SPR assays, were found to bind NadR with K_D_ values of 1.5 mM and 1.1 mM, respectively. Although these NadR/HPA interactions appeared rather weak, their distinct affinities and specificities matched their *in vitro* effects [9, 20] and their biological relevance appears similar to previous proposals that certain small molecules, including some antibiotics, in the millimolar concentration range may be broad inhibitors of MarR family proteins [13, 17]. Indeed, 4-HPA is found in human saliva [19] and 3Cl,4-HPA is produced during inflammatory processes [28], suggesting that these natural ligands are encountered by *N*. *meningitidis* in the mucosa of the oropharynx during infections. It is also possible that NadR responds to currently unidentified HPA analogues. Indeed, in the NadR/4-HPA complex there was a water molecule close to the carboxylate group and also a small unfilled tunnel ~5Å long, both factors suggesting that alternative larger ligands could occupy the pocket. It is conceivable that such putative ligands may establish different bonding networks, potentially binding in a 2:2 ratio, rather than the 1:2 ratio observed herein. The ability to respond to various ligands might enable NadR *in vivo* to orchestrate multiple response mechanisms and modulate expression of genes other than *nadA*. Ultimately, confirmation of the relevance of each ligand will require a deeper understanding of the available concentration *in vivo* in the host niche during bacterial colonization and inflammation.

Here, we determined the first crystal structures of apo-NadR and holo-NadR. These experimentally-determined structures enabled a new detailed characterization of the ligand-binding pocket. In holo-NadR, 4-HPA interacted directly with at least 11 polar and hydrophobic residues. Several, but not all, of these interactions were predicted previously by homology modelling combined with ligand docking *in silico* [21]. Subsequently, we established the functional importance of His7, Ser9, Asn11 and Phe25 in the *in vitro* response of meningococcus to 4-HPA, via site-directed mutagenesis. More unexpectedly, the crystal structure revealed that only one molecule of 4-HPA was bound per NadR dimer. We confirmed this stoichiometry in solution using SPR methods. We also used heteronuclear NMR spectroscopy to detect substantial conformational changes of NadR occurring in solution upon addition of 4-HPA. Moreover, NMR spectra at 10°C suggested the existence of two distinct conformations of NadR in the vicinity of the ligand-binding pocket. More powerfully, our unique crystallographic observation of this ‘occupied vs unoccupied site’ asymmetry in the NadR/4-HPA interaction is, to our knowledge, the first example reported for a MarR family protein. Structural analyses suggested that ‘inward’ side chain positions of Met22, Phe25 and especially Arg43 precluded binding of a second ligand molecule. Such a mechanism indicates negative cooperativity, which may enhance the ligand-responsiveness of NadR.

**Fig 10:**
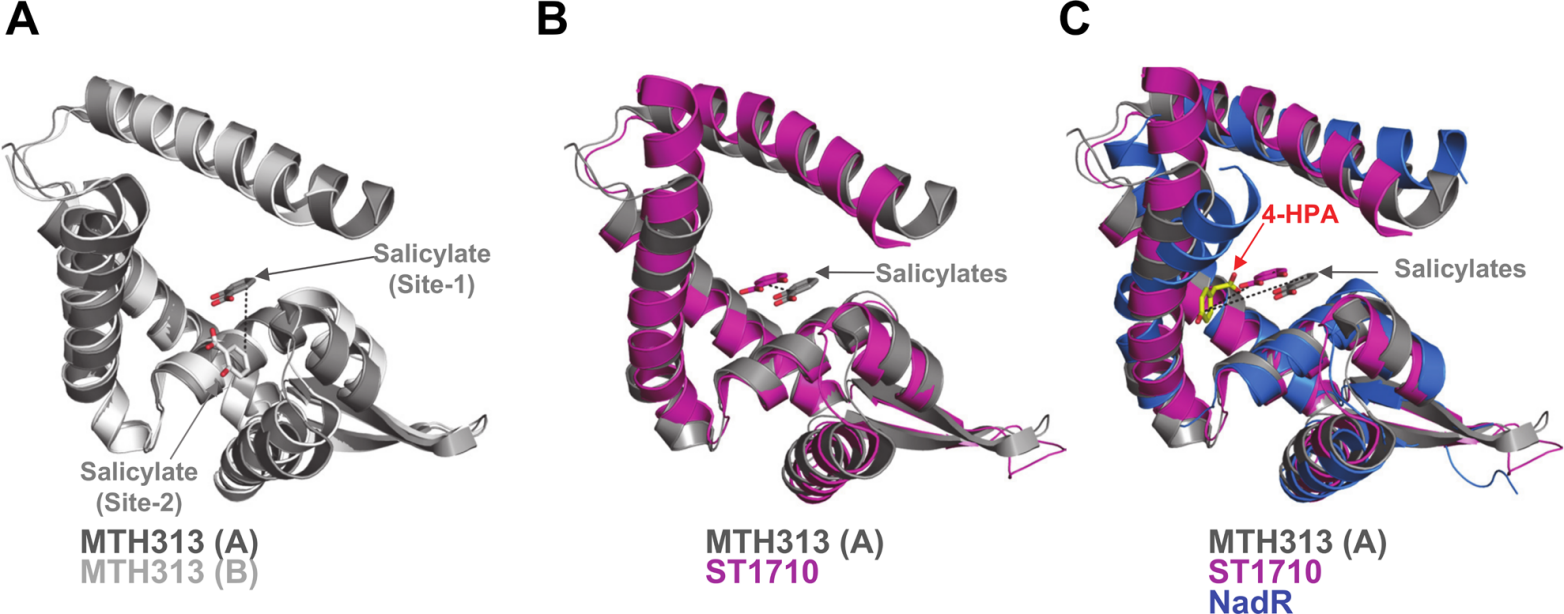
NadR shows a ligand binding site distinct from other MarR homologues. **(A)** A structural alignment of MTH313 chains A and B shows that salicylate is bound in distinct locations in each monomer; site-1 (thought to be the biologically relevant site) and site-2 differ by ~7Å (indicated by black dotted line) and also by ligand orientation. **(B)** A structural alignment of MTH313 chain A and ST1710 (pink) (Cα rmsd 2.3Å), shows that they bind salicylate in equivalent sites (differing by only ~3Å) and with the same orientation. **(C)** Addition of holo-NadR (chain B, blue) to the alignment reveals that bound 4-HPA differs in position by > 10 Å compared to salicylate, and adopts a novel orientation.

Comparisons of the NadR/4-HPA complex with available MarR family/salicylate complexes revealed that 4-HPA has a previously unobserved binding mode. Briefly, in the *M*. *thermoautotrophicum* MTH313 dimer, one molecule of salicylate binds in the pocket of each monomer, though with two rather different positions and orientations, only one of which (site-1) is thought to be biologically relevant [17] (Fig 10A). In the *S*. *tokodaii* protein ST1710, salicylate binds to the same position in each monomer of the dimer, in a site equivalent to the putative biologically relevant site of MTH313 [18] (Fig 10B). Unlike other MarR family proteins which revealed multiple ligand binding interactions, we observed only 1 molecule of 4-HPA bound to NadR, suggesting a more specific and less promiscuous interaction. In NadR, the single molecule of 4-HPA binds in a position distinctly different from the salicylate binding site: translated by > 10 Å and with a 180° inverted orientation (Fig 10C).

Interestingly, a crystal structure was previously reported for a functionally-uncharacterized meningococcal homologue of NadR, termed NMB1585, which shares 16% sequence identity with NadR [29]. The two structures can be closely aligned (rmsd 2.3 Å), but NMB1585 appears unsuited for binding HPAs, since its corresponding ‘pocket’ region is occupied by several bulky hydrophobic side chains. It can be speculated that MarR family members have evolved separately to engage distinct signaling molecules, thus enabling bacteria to use the overall conserved MarR scaffold to adapt and respond to diverse changing environmental stimuli experienced in their natural niches. Alternatively, it is possible that other MarR homologues (e.g. NMB1585) may have no extant functional binding pocket and thus may have lost the ability to respond to a ligand, acting instead as constitutive DNA-binding regulatory proteins.

The apo-NadR crystal structures revealed two dimers with slightly different conformations, most divergent in the DNA-binding domain. It is not unusual for a crystal structure to reveal multiple copies of the same protein in very slightly different conformations, which are likely representative of the lowest-energy conformations sampled by the dynamic ensemble of molecular states occurring in solution, and which likely have only small energetic differences, as described previously for MexR (a MarR protein) [30] or more recently for the solute-binding protein FhuD2 [31, 32]. Further, the holo-NadR structure was overall more different from the two apo-NadR structures (rmsd values ~1.3Å), suggesting that the ligand selected and stabilized yet another conformation of NadR. These observations suggest that 4-HPA, and potentially other similar ligands, can shift the molecular equilibrium, changing the energy barriers that separate active and inactive states, and stabilizing the specific conformation of NadR poorly suited to bind DNA.

Comparisons of the apo- and holo-NadR structures revealed that the largest differences occurred in the DNA-binding helix α4. The shift of helix α4 in holo-NadR was also accompanied by rearrangements at the dimer interface, involving helices α1, α5, and α6, and this holo-form appeared poorly suited for DNA-binding when compared with the known OhrR:*ohrA* complex [26]. While some flexibility of helix α4 was also observed in the two apo-structures, concomitant changes in the dimer interfaces were not observed, possibly due to the absence of ligand. One of the two conformations of apo-NadR appeared ideally suited for DNA-binding. Overall, these analyses suggest that the apo-NadR dimer has a pre-existing equilibrium that samples a variety of conformations, some of which are compatible with DNA binding. This intrinsically dynamic nature underlies the possibility for different conformations to inter-convert or to be preferentially selected by a regulatory ligand, as generally described in the ‘conformational selection’ model for protein-ligand interactions (the Monod-Wyman-Changeux model), rather than an ‘induced fit’ model (Koshland-Nemethy-Filmer) [33]. The noted flexibility may also explain how NadR can adapt to bind various DNA target sequences [10] with slightly different structural features. Subsequently, upon ligand binding, holo-NadR adopts a structure less suited for DNA-binding and this conformation is selected and stabilized by a network of protein-ligand interactions and concomitant rearrangements at the NadR holo dimer interface. In an alternative and less extensive manner, the binding of two salicylate molecules to the *M*. *thermoautotrophicum* protein MTH313 appeared to induce large changes in the wHTH domain, which was associated with reduced DNA-binding activity [17].

Here we have presented two new crystal structures of the transcription factor, NadR, which regulates expression of the meningococcal surface protein, virulence factor and vaccine antigen NadA. Detailed structural analyses provided a molecular explanation for the ligand-responsive regulation by NadR on the majority of the promoters of meningococcal genes regulated by NadR, including *nadA* [10]. Intriguingly, NadR exhibits a reversed regulatory mechanism on a second class of promoters, including *mafA* of the multiple adhesin family–i.e. NadR represses these genes in the presence but not absence of 4-HPA. The latter may influence the surface abundance or secretion of *maf* proteins, an emerging class of highly conserved meningococcal putative adhesins and toxins with many important roles [11, 12]. Further work is required to investigate how the two different promoter types influence the ligand-responsiveness of NadR during bacterial infection and may provide insights into the regulatory mechanisms occurring during these host-pathogen interactions. Ultimately, knowledge of the ligand-dependent activity of NadR will continue to deepen our understanding of *nadA* expression levels, which influence meningococcal pathogenesis.

## Materials and Methods

### Bacterial strains, culture conditions and mutant generation

In this study we used *N*. *meningitidis* MC58 wild type strain and related mutant derivatives. The MC58 isolate was kindly provided to us by Professor E. Richard Moxon, University of Oxford, UK, and was previously submitted to the Meningococcal Reference Laboratory, Manchester, UK [34]. Strains were routinely cultured, stocked, and transformed as described previously [10]. To generate *N*. *meningitidis* MC58 mutant strains expressing only the amino acid substituted forms of NadR, plasmids containing the sequence of *nadR* mutated to insert alanine codons to replace His7, Ser9, Asn11 or Phe25 were constructed using the QuikChange II XL Site-Directed Mutagenesis Kit (Stratagene). The *nadR* gene (also termed NMB1843) was mutated in the pComEry-1843 plasmid using couples of mutagenic primers (forward and reverse). The resulting plasmids were named pComEry-1843H7A, -1843S9A, -1843N11A or -1843F25A, and contained a site-directed mutant allele of the *nadR* gene, in which the selected codons were respectively substituted by a GCG alanine codon, and were used for transformation of the MC-Δ1843 strain. Total lysates from single colonies of all transformants were used as a template for PCR analysis to confirm the correct insertion by double homologous recombinant event. When indicated, bacterial strains were grown in presence of 5 mM 4-HPA (MW 152, Sigma-Aldrich).

### Molecular cloning

The preparation of the expression construct enabling production of soluble NadR with an N-terminal His-tag followed by a thrombin cleavage site (MGSSHHHHHHSSGLVPR↓GSH-) (where the arrow indicates the cleavage site) and then NadR residues M1-S146 (Uniprot code Q7DD70), and methods to generate site-directed mutants, were described previously [21]. Briefly, site-directed mutagenesis was performed using two overlapping primers containing the desired mutation to amplify pET15b containing several NadR variants. (Full oligonucleotide sequences of primers are available upon request). 1–10 ng of plasmid DNA template were amplified using Kapa HiFi DNA polymerase (Kapa Biosystems) and with the following cycling conditions: 98°C for 5 min, 15 cycles of (98°C for 30 s, 60°C for 30 s, 72°C for 6 min) followed by a final extension of 10 min at 72°C. Residual template DNA was digested by 30 min incubation with FastDigest DpnI (Thermo Scientific) at 37°C and 1 μl of this reaction was used to transform *E*. *coli* DH5α competent cells. The full recombinant tagged NadR protein generated contained 166 residues, with a theoretical MW of 18746, while after thrombin-cleavage the untagged protein contained 149 residues, with a theoretical MW of 16864.

### Protein production and purification

The NadR expression constructs (wild-type or mutant clones) were transformed into *E*. *coli* BL21 (DE3) cells and were grown at 37°C in Luria-Bertani (LB) medium supplemented with 100 μg/mL ampicillin, until an OD_600_ of 0.5 was reached. Target protein production was induced by the addition of 1 mM IPTG followed by incubation with shaking overnight at 21°C. For production of the selenomethionine (SeMet) derivative form of NadR for crystallization studies, essentially the same procedure was followed, but using the *E*. *coli* B834 strain grown in a modified M9 minimal medium supplemented with 40 mg/L L-SeMet. For production of ^15^N-labeled NadR for NMR analyses, the EnPresso B Defined Nitrogen-free medium (Sigma-Aldrich) was used; in brief, BL21 (DE3) cells were grown in BioSilta medium at 30°C for 30 h, and production of the ^15^N-labeled NadR was enabled by the addition of 2.5 g/L ^15^NH_4_Cl and further incubation for 2 days.

Cells were harvested by centrifugation (6400 g, 30 min, 4°C), resuspended in 20 mM HEPES pH 8.0, 300 mM NaCl, 20 mM imidazole, and were lysed by sonication (Qsonica Q700). Cell lysates were clarified by centrifugation at 2800 g for 30 min, and the supernatant was filtered using a 0.22 μm membrane (Corning filter system) prior to protein purification. NadR was purified by affinity chromatography using an AKTA purifier (GE Healthcare). All steps were performed at room temperature (18–26°C), unless stated otherwise. The filtered supernatant was loaded onto an Ni-NTA resin (5 mL column, GE Healthcare), and NadR was eluted using 4 steps of imidazole at 20, 30, 50 and 250 mM concentration, at a flow rate of 5 mL/min. Eluted fractions were examined by reducing and denaturing SDS-PAGE analysis. Fractions containing NadR were identified by a band migrating at ~17 kDa, and were pooled. The N-terminal 6-His tag was removed enzymatically using the Thrombin CleanCleave Kit (Sigma-Aldrich). Subsequently, the sample was reloaded on the Ni-NTA resin to capture the free His tag (or unprocessed tagged protein), thus allowing elution in the column flow-through of tagless NadR protein, which was used in all subsequent studies. The NadR sample was concentrated and loaded onto a HiLoad Superdex 75 (16/60) preparative size-exclusion chromatography (SEC) column equilibrated in buffer containing 20 mM HEPES pH 8.0, 150 mM NaCl, at a flow-rate of 1 mL/min. NadR protein was collected and the final yield of purified protein obtained from 0.5 L LB growth medium was approximately 8 mg (~2 mg protein per g wet biomass). Samples were used immediately for crystallization or analytical experiments, or were frozen for storage at -20°C.

### SE-HPLC/MALLS analyses

MALLS analyses were performed online with SE-HPLC using a Dawn TREOS MALLS detector (Wyatt Corp., Santa Barbara, CA, USA) and an incident laser wavelength of 658 nm. The intensity of the scattered light was measured at 3 angles simultaneously. Data analysis was performed using the Astra V software (Wyatt) to determine the weighted-average absolute molecular mass (MW), the polydispersity index (MW/Mn) and homogeneity (Mz/Mn) for each oligomer present in solution. Normalization of the MALLS detectors was performed in each analytical session by use of bovine serum albumin.

### Differential scanning calorimetry

The thermal stability of NadR proteins was assessed by differential scanning calorimetry (DSC) using a MicroCal VP-Capillary DSC instrument (GE Healthcare). NadR samples were prepared at a protein concentration of 0.5 mg/mL (~30 μM) in buffer containing 20 mM HEPES, 300 mM NaCl, pH 7.4, with or without 6 mM HPA or salicylate. The DSC temperature scan ranged from 10°C to 110°C, with a thermal ramping rate of 200°C per hour and a 4 second filter period. Data were analyzed by subtraction of the reference data for a sample containing buffer only, using the Origin 7 software. All experiments were performed in triplicate, and mean values of the melting temperature (T_m_) were determined.

### Surface plasmon resonance (SPR)

#### Determination of equilibrium dissociation constant, K_D_

Surface plasmon resonance binding analyses were performed using a Biacore T200 instrument (GE Healthcare) equilibrated at 25°C. The ligand (NadR) was covalently immobilized by amine-coupling on a CM-5 sensor chip (GE Healthcare), using 20 μg/mL purified protein in 10 mM sodium acetate buffer pH 5, injected at 10 μl/min for 120 s until ~9000 response units (RU) were captured. A high level of ligand immobilization was required due to the small size of the analytes. An unmodified surface was used as the reference channel. Titrations with analytes (HPAs or salicylate) were performed with a flow-rate of 30 μl/min, injecting the compounds in a concentration range of 10 μM to 20 mM, using filtered running buffer containing Phosphate Buffered Saline (PBS) with 0.05% Tween-20, pH 7.4. Following each injection, sensor chip surfaces were regenerated with a 30-second injection of 10 mM Glycine pH 2.5. Each titration series contained 20 analyte injections and was performed in triplicate. Titration experiments with long injection phases (> 15 mins) were used to enable steady-state analyses. Data were analyzed using the BIAcore T200 evaluation software and the steady-state affinity model. A buffer injection was subtracted from each curve, and reference sensorgrams were subtracted from experimental sensorgrams to yield curves representing specific binding. The equilibrium dissociation constant, K_D_, was determined from the plot of RU_eq_ against analyte concentration (S2 Fig), as described previously [35]. Determination of binding stoichiometry: From each plot of RU_eq_ against analyte concentration, obtained from triplicate experiments, the R_max_ value (maximum analyte binding capacity of the surface) was extrapolated from the experimental data (S2 Fig). Stoichiometry was calculated using the molecular weight of dimeric NadR as ligand molecule (MW_ligand_) and the molecular weights of the HPA analyte molecules (MW_analyte_), and the following equation:

$$Stoichiometry=\frac{{R_{max}\times MW}_{ligand}}{{MW}_{analyte}\times R_{ligand}}$$ (1)

where R_ligand_ is recorded directly from the sensorgram during ligand immobilization prior to the titration series, as described previously [24]. The stoichiometry derived therefore represented the number of HPA molecules bound to one dimeric NadR protein.

### Crystallization of NadR in the presence or absence of 4-HPA

Purified NadR was concentrated to 2.7 mg/mL (~160 μM) using a centrifugal concentration device (Amicon Ultra-15 Centrifugal Filter Unit with Ultracel-10 membrane with cut-off size 10 kDa; Millipore) running at 600 *g* in a bench top centrifuge (Thermo Scientific IEC CL40R) refrigerated at 2–8°C. To prepare holo-NadR samples, HPA ligands were added at a 200-fold molar excess prior to the centrifugal concentration step. The concentrated holo- or apo-NadR was subjected to crystallization trials performed in 96-well low-profile Intelli-Plates (Art Robbins) or 96-well low-profile Greiner crystallization plates, using a nanodroplet sitting-drop vapour-diffusion format and mixing equal volumes (200 nL) of protein samples and crystallization buffers using a Gryphon robot (Art Robbins). Crystallization trays were incubated at 20°C. Crystals of apo-NadR were obtained in 50% PEG 3350 and 0.13 M di-Ammonium hydrogen citrate, whereas crystals of SeMet–NadR in complex with 4-HPA grew in condition H4 of the Morpheus screen (Molecular Dimensions), which contains 37.5% of the pre-mixed precipitant stock MPD_P1K_PEG 3350, buffer system 1 and 0.1 M amino acids, at pH 6.5. All crystals were mounted in cryo-loops using 10% ethylene glycol or 10% glycerol as cryo-protectant before cooling to 100 K for data collection.

### X-ray diffraction data collection and structure determination

X-ray diffraction data from crystals of apo-NadR and SeMet–NadR/4-HPA were collected on beamline PXII-X10SA of the Swiss Light Source (SLS) at the Paul Scherrer Institut (PSI), Villigen, Switzerland. All diffraction data were processed with *XDS* [36] and programs from the CCP4 suite [37]. Crystals of apo-NadR and 4-HPA-bound SeMet-NadR belonged to space group *P*43 21 2 (see Table 2). Apo-NadR crystals contained four molecules (two dimers) in the asymmetric unit (Matthews coefficient 2.25 Å^3^ Da^−1^, for a solvent content of 45%), while crystals of SeMet–NadR/4-HPA contained two molecules (one dimer) in the asymmetric unit (Matthews coefficient 1.98 Å^3^ Da^−1^, for a solvent content of 38%). In solving the holo-NadR structure, an initial and marginal molecular replacement (MR) solution was obtained using as template search model the crystal structure of the transcriptional regulator PA4135 (PBD entry 2FBI), with which NadR shares ~54% sequence identity. This solution was combined with SAD data to aid identification of two selenium sites in NadR, using *autosol* in *phenix* [38] and this allowed generation of high-quality electron density maps that were used to build and refine the structure of the complex. Electron densities were clearly observed for almost the entire dimeric holo-NadR protein, except for a new N-terminal residues and residues 88–90 of chain B.

The crystal structure of apo-NadR was subsequently solved by MR in *Phaser* [39] at 2.7 Å, using the final refined model of SeMet-NadR/4-HPA as the search model. For apo-NadR, electron densities were clearly observed for almost the entire protein, although residues 84–91 of chains A, C, and D, and residues 84–90 of chain B lacked densities suggesting local disorder.

Both structures were refined and rebuilt using *phenix* [38] and *Coot* [40], and structural validation was performed using Molprobity [41]. Data collection and refinement statistics are reported in Table 2. Atomic coordinates of the two NadR structures have been deposited in the Protein Data Bank, with entry codes 5aip (NadR bound to 4-HPA) and 5aiq (apo-NadR). All crystallographic software was compiled, installed and maintained by SBGrid [42].

### NMR spectroscopy

For heteronuclear NMR experiments, the NadR protein concentration used was 85 μM (~ 1.4 mg/mL) in a solution containing 100 mM sodium phosphate buffer (90% H_2_O and 10% D_2_O) and 200 mM NaCl, prepared in the apo-form or in the presence of a 200-fold molar excess of 4-HPA, at pH 6.5. The stability, integrity and dimeric state of the protein in the NMR buffer was confirmed by analytical SEC (Superdex 75, 10/300 column) prior to NMR studies. ^1^H-^15^N transverse relaxation-optimized spectroscopy (TROSY)-heteronuclear single quantum coherence (HSQC) experiments on apo-NadR and NadR in the presence of 4-HPA were acquired using an Avance 950 Bruker spectrometer, operating at a proton frequency of 949.2 MHz and equipped with triple resonance cryogenically-cooled probe at two different temperatures (298 K and 283 K). ^1^H-^15^N TROSY-HSQC experiments were recorded for 12 h, with a data size of 1024 x 232 points. Spectra were processed using the Bruker TopSpin 2.1 and 3.1 software packages.

### Western blot

Western blot analysis was performed as described previously [10].

## Supporting Information

S1 Fig Absolute molecular mass of NadR. Multi-angle laser light scattering **(**MALLS) analyses were performed to determine the absolute molecular mass of NadR alone **(A)** or in the presence of 4-HPA ligand **(B)**. The curves plotted correspond to Absorbance Units (mAU) at 280nm wavelength (green), light scattering (red), and refractive index (blue). The elution peak maxima were at 17.5 minutes and the numerical data obtained for absolute molecular mass and polydispersity are shown below each image. In both cases, the MALLS data clearly indicated a single monodisperse species of absolute molecular mass ~ 37.5 kDa, corresponding to the dimeric form of NadR. (The numbers ‘1’ at the bottom of the gradient-shaded slice identify the beginning and end of each fraction-1, used for the MALLS analyses).
(TIF)

S2 Fig Studies of the NadR-ligand interactions. Surface plasmon resonance (SPR) was used to determine the equilibrium dissociation constants (K_D_), using the steady-state approach, for the ligands 4-HPA **(A)**, salicylate **(B)**, 3Cl,4-HPA **(C)** and 3-HPA **(D)**. SPR data are shown as equilibrium binding response (RU_eq_) plotted against analyte (HPA) concentration (mM). Each data point shown represents the mean RU_eq_ value from three replicate experiments, as described in the main text *Materials & Methods* section. R_max_ values for each curve are indicated; n.d.: not determinable. The titrations included ligand concentrations from 10μM to 20mM. For 3-HPA and 4-HPA, all data points were used for curve-fitting; while for 3Cl,4-HPA, data points at analyte concentration > 4mM were excluded due to non-specific association of the analyte on the sensor chip surface, possibly due to lower solubility of this compound. As reported in the main text, the plot of equilibrium binding response (RU_eq_) against analyte concentration enabled determination of the equilibrium binding constants (K_D_) via the steady-state approach, and determination of R_max_ enabled calculation of the binding stoichiometries (except for salicylate where R_max_ could not be reliably determined).
(TIF)

S3 Fig Conformational differences between apo- and holo-NadR structures. **(A)** Apo_AB_ vs Holo; **(B)** Apo_AB_ vs Apo_CD_. For clarity, the comparisons are shown side-by-side with dimers ‘pulled apart horizontally’. The major rearrangement of the DNA-binding helix α4 observed when comparing apo chain B (pale blue) and holo chain B (blue) **(A, right)** is concomitant with notable changes (indicated by curvy arrows) in helices α1, α5, and α6, on both sides of the dimer interface, which depart from an optimal structural alignment in both pairs of superposed monomers. Instead, the two apo-homodimers mainly differ only in the orientation of the region α2-α5 and only in their B chains (**B, right**), while the α6 helices at the dimer interface were much less different (compare the alignments of helix α6 in **A** and **B**).
(TIF)
