# Supplementary figures and images for: Molecular Basis of Ligand-Dependent Regulation of NadR, the Transcriptional Repressor of Meningococcal Virulence Factor NadA
Source: PLoS Pathog. 2016 Apr 22;12(4):e1005557. doi: 10.1371/journal.ppat.1005557 (PMC4841544; doi:10.1371/journal.ppat.1005557)

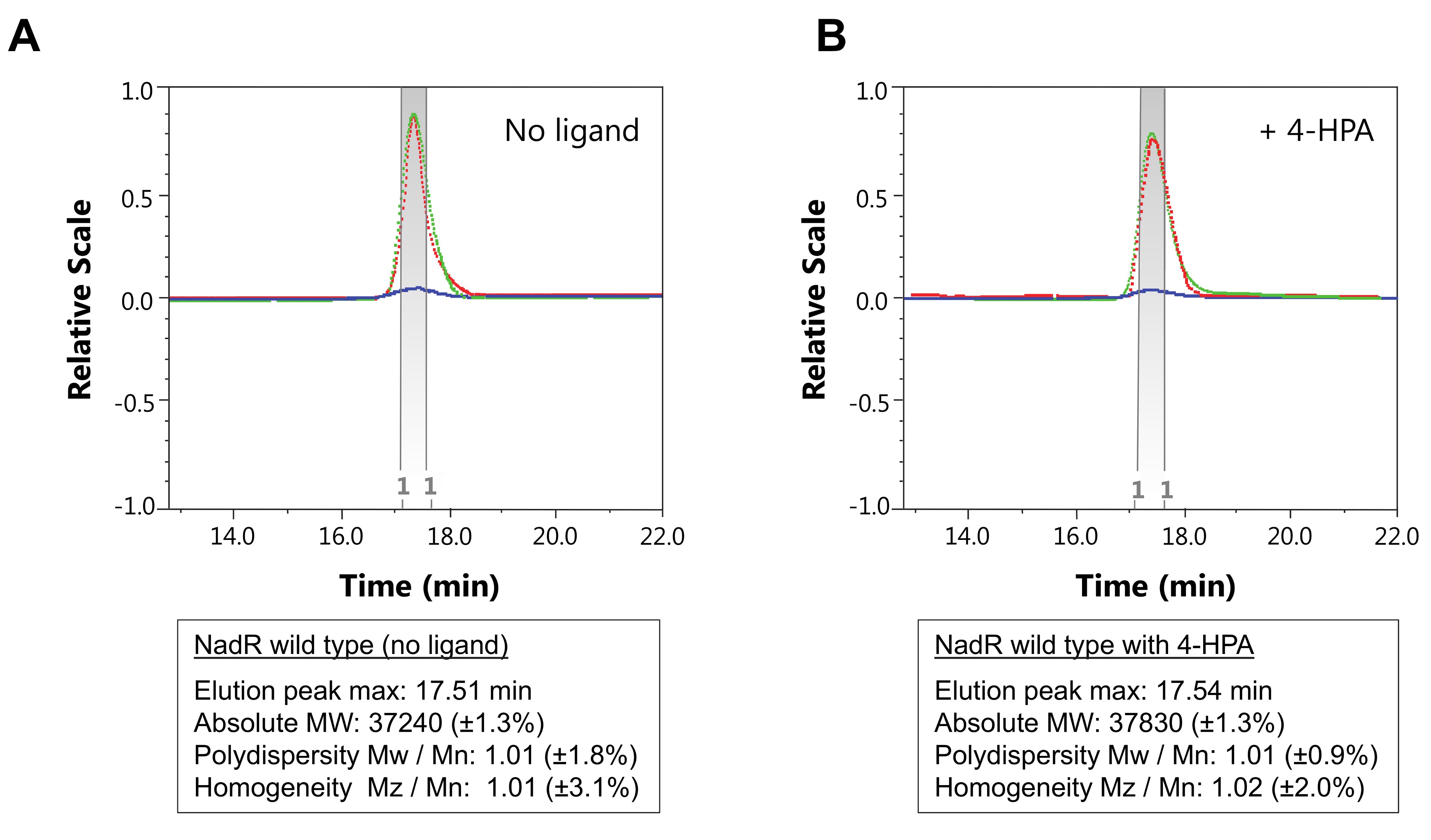

Supplement: S1 Fig — Multi-angle laser light scattering (MALLS) analyses were performed to determine the absolute molecular mass of NadR alone (A) or in the presence of 4-HPA ligand (B). The curves plotted correspond to Absorbance Units (mAU) at 280nm wavelength (green), light scattering (red), and refractive index (blue). The elution peak maxima were at 17.5 minutes and the numerical data obtained for absolute molecular mass and polydispersity are shown below each image. In both cases, the MALLS data clearly indicated a single monodisperse species of absolute molecular mass ~ 37.5 kDa, corresponding to the dimeric form of NadR. (The numbers ‘1’ at the bottom of the gradient-shaded slice identify the beginning and end of each fraction-1, used for the MALLS analyses). (TIF) [file ppat.1005557.s001.tif]

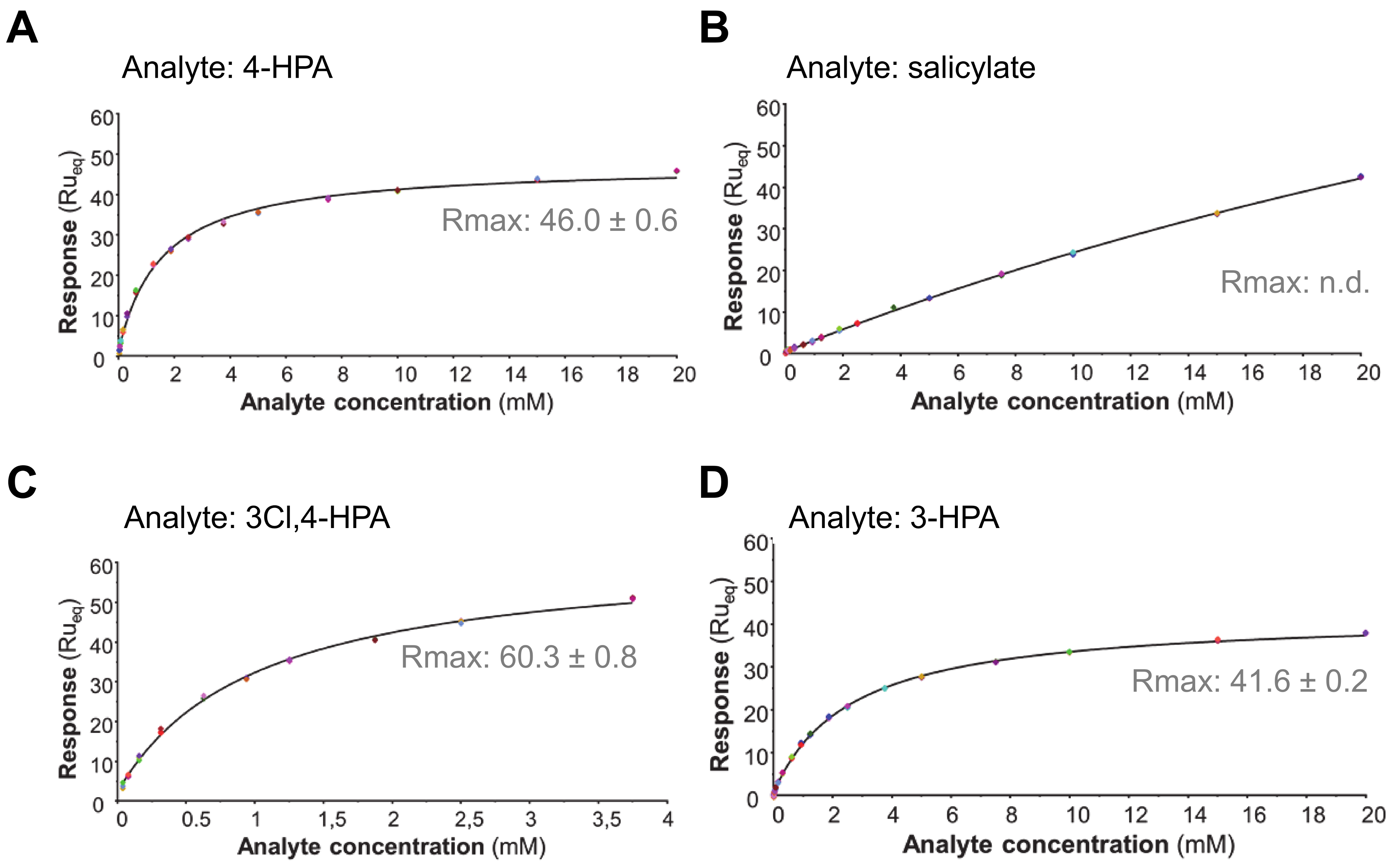

Supplement: S2 Fig — Surface plasmon resonance (SPR) was used to determine the equilibrium dissociation constants (KD), using the steady-state approach, for the ligands 4-HPA (A), salicylate (B), 3Cl,4-HPA (C) and 3-HPA (D). SPR data are shown as equilibrium binding response (RUeq) plotted against analyte (HPA) concentration (mM). Each data point shown represents the mean RUeq value from three replicate experiments, as described in the main text Materials & Methods section. Rmax values for each curve are indicated; n.d.: not determinable. The titrations included ligand concentrations from 10μM to 20mM. For 3-HPA and 4-HPA, all data points were used for curve-fitting; while for 3Cl,4-HPA, data points at analyte concentration > 4mM were excluded due to non-specific association of the analyte on the sensor chip surface, possibly due to lower solubility of this compound. As reported in the main text, the plot of equilibrium binding response (RUeq) against analyte concentration enabled determination of the equilibrium binding constants (KD) via the steady-state approach, and determination of Rmax enabled calculation of the binding stoichiometries (except for salicylate where Rmax could not be reliably determined). (TIF) [file ppat.1005557.s002.tif]

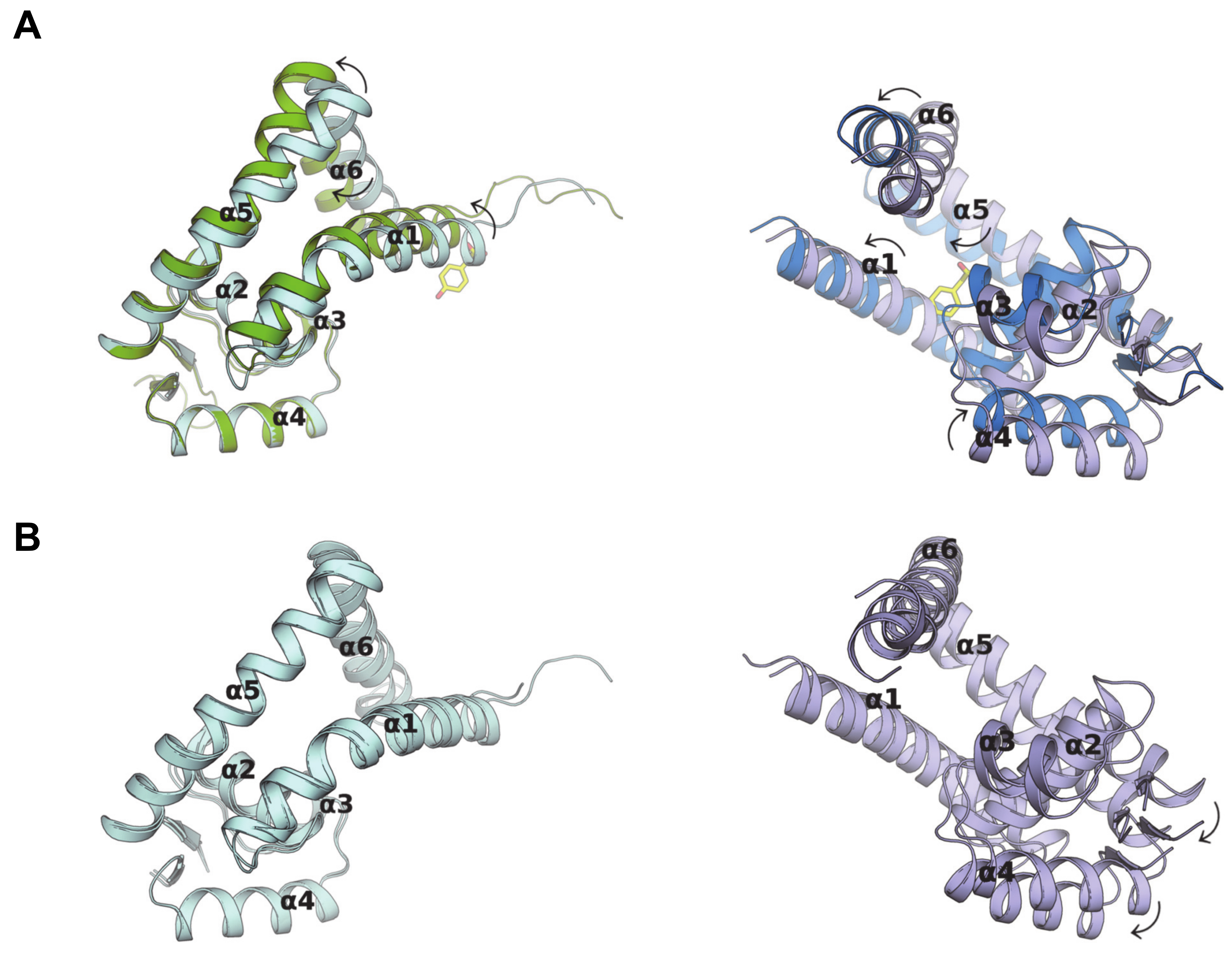

Supplement: S3 Fig — (A) ApoAB vs Holo; (B) ApoAB vs ApoCD. For clarity, the comparisons are shown side-by-side with dimers ‘pulled apart horizontally’. The major rearrangement of the DNA-binding helix α4 observed when comparing apo chain B (pale blue) and holo chain B (blue) (A, right) is concomitant with notable changes (indicated by curvy arrows) in helices α1, α5, and α6, on both sides of the dimer interface, which depart from an optimal structural alignment in both pairs of superposed monomers. Instead, the two apo-homodimers mainly differ only in the orientation of the region α2-α5 and only in their B chains (B, right), while the α6 helices at the dimer interface were much less different (compare the alignments of helix α6 in A and B). (TIF) [file ppat.1005557.s003.tif]
